# Optimizing the maximum reported cluster size in the spatial scan statistic for survival data

**DOI:** 10.1186/s12942-021-00286-w

**Published:** 2021-07-08

**Authors:** Sujee Lee, Jisu Moon, Inkyung Jung

**Affiliations:** Division of Biostatistics, Department of Biomedical Systems Informatics, Yonsei University College of Medicine, 50-1 Yonsei-ro, Seodaemun-gu, Seoul, 03722 Korea

**Keywords:** Spatial cluster detection, Exponential model, Gini coefficient, SaTScan

## Abstract

**Background:**

The spatial scan statistic is a useful tool for cluster detection analysis in geographical disease surveillance. The method requires users to specify the maximum scanning window size or the maximum reported cluster size (MRCS), which is often set to 50% of the total population. It is important to optimize the maximum reported cluster size, keeping the maximum scanning window size at as large as 50% of the total population, to obtain valid and meaningful results.

**Results:**

We developed a measure, a Gini coefficient, to optimize the maximum reported cluster size for the exponential-based spatial scan statistic. The simulation study showed that the proposed method mostly selected the optimal MRCS, similar to the true cluster size. The detection accuracy was higher for the best chosen MRCS than at the default setting. The application of the method to the Korea Community Health Survey data supported that the proposed method can optimize the MRCS in spatial cluster detection analysis for survival data.

**Conclusions:**

Using the Gini coefficient in the exponential-based spatial scan statistic can be very helpful for reporting more refined and informative clusters for survival data.

**Supplementary Information:**

The online version contains supplementary material available at 10.1186/s12942-021-00286-w.

## Background

The spatial scan statistic is a useful and widely used tool for detecting spatial or space–time clusters in disease surveillance. The method has been developed for different types of data such as count [1], ordinal [2, 3], survival [4], continuous [5–7], and multinomial [8]. The software SaTScan™ [9], available for free, enhances the ease of access to this method for researchers.

The spatial scan statistic is formulated based on the likelihood ratio test statistic. A large number of scanning windows of various sizes across all locations are first constructed on the entire study area. Each scanning window is a candidate for the most likely cluster. In SaTScan™, circular or elliptical scanning windows are considered. The likelihood ratio test statistic is calculated for each window to compare its inside and outside. The scanning window with the maximum value of the likelihood ratio test statistic is defined as the most likely cluster. Secondary clusters with high test statistic values are also reported.

Cluster detection results can be sensitive to the maximum scanning window size (MSWS), as studied by Riberiro and Costa [10]. In SaTScan™, users can specify the MSWS, which is set to 50% of the total population by default. A high MSWS and a high maximum reported cluster size (MRCS) could result in an excessively large cluster. Some researchers try different MSWS values to obtain seemingly good results without knowing the MRCS. Repeatedly performing spatial cluster detection analyses using different values of MSWS leads to a multiple testing problem, as pointed out by Han et al. [11]. We can consider different values of MRCS with a fixed MSWS to avoid this problem. Still, we need to choose the optimal value of the MRCS. The clusters reported by subjectively chosen MRCS may be different from the true clusters.

Han et al. [11] proposed a criterion measure to optimize the MRCS for the Poisson-based spatial scan statistic. They defined the Gini coefficient to represent the degree of heterogeneity of disease clusters for count data. Their simulation study showed that the Gini coefficient can be useful for selecting the best MRCS to obtain a refined collection of clusters. Interestingly, by reporting an optimized and refined collection of clusters rather than a single large cluster, the Gini coefficient allows us to better identify irregularly shaped ones [12].

As the formulation of test statistics of the spatial scan statistic is different for different models, the Gini coefficient should be clearly and distinctly defined for each model and thoroughly evaluated. The Gini coefficients for the ordinal- and normal-based spatial scan statistics were proposed by Kim and Jung [13] and by Yoo and Jung [14], respectively. In this paper, we defined the Gini coefficient for the exponential-based spatial scan statistic, which is used for survival data. Through an extensive simulation study under various scenarios, we showed that the proposed method is very useful for optimizing the MRCS for the exponential-based spatial scan statistic. We illustrated the method using Community Health Survey data collected by the Korea Centers for Disease Control and Prevention.

## Methods

### Poisson model and the Gini coefficient

When we have count data such as the number of certain disease occurrences according to an underlying population at risk in a study region, we can use the Poisson-based spatial scan statistic [1]. We are often interested in identifying areas with high disease incidence rates. The null and alternative hypotheses are written as

$${H_0}:p = q\;{\text{for all}}\;z \in Z\;vs.\;{H_a}:p > q\;{\text{for some}}\;z \in Z$$

where *p* and *q* are the intensities of the outcome variable inside and outside the scanning window $z$, respectively, and *Z* denotes the collection of all scanning windows. The likelihood ratio test statistic given window $z$ is expressed as

$$LR\left(z\right)=\frac{{\left(\frac{{c}_{z}}{{n}_{z}}\right)}^{{c}_{z}}{\left(\frac{C-{c}_{z}}{N-{n}_{z}}\right)}^{{C-c}_{z}}}{{\left(\frac{C}{N}\right)}^{C}}$$

if ${c}_{z}/{n}_{z}>({C-c}_{z})/({N-n}_{z})$, and $LR\left(z\right)=1$ otherwise. In the above equation, ${c}_{z}$and ${n}_{z}$ denote the observed number of cases and population within window *z*. $C$ and $N$ denote the total number of cases and population in the whole study area, respectively.

The scanning window that maximizes the value of $LR\left(z\right)$ is the most likely cluster. Statistical inference for the most likely cluster can be performed using Monte Carlo hypothesis testing. In addition, secondary clusters with high values of the likelihood ratio test statistic are often of interest. The p-values of the secondary clusters are typically obtained in the same manner as the null hypothesis is rejected on own strength.

When reporting the most likely and secondary clusters, the Gini coefficient can be used to find a more refined collection of non-overlapping clusters. In economics, the Gini coefficient was developed to indicate the degree of heterogeneity of wealth distribution [15]. As a summary measure of the Lorenz curve, the larger the Gini coefficient, the higher the heterogeneity in wealth. Han et al. [11] adopted the Gini coefficient in the spatial scan statistic for count data to measure the degree of heterogeneity in the spatial distribution of disease cases by defining the x-axis of the Lorenz curve as the cumulative proportion of the number of disease cases and the y-axis as the cumulative proportion of the population. Its value is calculated as twice the area between the Lorenz curve and the 45° line, which indicates that the number of cases is proportional to the population of each region. When there is only one significant cluster, the Lorenz curve is constructed as a line graph connecting the three points (0,0), (${x}_{1},{y}_{1}$), and (1,1), where ${x}_{1}$ and ${y}_{1}$ are the proportions of observed cases and population (expected cases) in the cluster. As more cases are concentrated in the cluster than expected, ${x}_{1}$ increases and the Lorenz curve moves farther away from the reference line. The Gini coefficient also increases. When we have *K* multiple clusters, the Lorenz curve connects *K* points between (0,0) and (1,1). The coordinates of each cluster $({x}_{k},{y}_{k})$ are defined as ${x_k} = \left( {\frac{1}{C}} \right)\mathop \sum \nolimits_{j = 1}^k {c_j}$ and ${y_k} = \left( {\frac{1}{N}} \right)\mathop \sum \nolimits_{j = 1}^k {n_j}$ where ${c}_{j}$ and ${n}_{j}$ are the number of cases and population in the $j$-th cluster. The Gini coefficient can be calculated as ${\sum }_{k=1}^{K+1}({y}_{k}{x}_{k-1}-{y}_{k-1}{x}_{k})$ with ${x}_{0}={y}_{0}=0$ and ${x}_{K+1}={y}_{K+1}=1.$ The Gini coefficient values range from 0 to 1. We select the best collection of clusters to report the highest Gini coefficient value from among several competing sets of clusters. Han et al. [11] included more detailed information. The Gini coefficient has been implemented in SaTScan™ for the Poisson and Bernoulli models.

### Spatial scan statistic for survival data

Different spatial scan statistics for survival data have been proposed based on different models, including Weibull and generalized life distributions [16, 17]. Huang et al. [4] proposed a spatial scan statistic for survival data based on an exponential model. We focused on the exponential model. The exponential-based spatial scan statistic has been used to examine geographic disparities in survival in cancer patients [18–20].

Suppose we have survival data for *I* subjects in a study area, such as time to death for cancer patients. Let ${T}_{i}$ and ${L}_{i}$ be the survival time and fixed censoring time for the $i$ th subject, respectively. We assume that ${T}_{i}$ is exponentially distributed with a probability density function $f\left( {{T_i}} \right) = \frac{1}{\theta }{e^{ - \frac{{{T_i}}}{\theta }}},\;\theta > 0.$ Parameter $\theta$ represents mean survival time. The observed time ${t_i} = \min \left( {{T_i},{L_i}} \right).$ Let ${\delta _i}$be the censoring indicator, that is, ${\delta _i} = 1{\text{ if }}{T_i} \leqslant {L_i}$ and ${\delta _i} = 0\;{\text{if }}{T_i} > {L_i}$ To identify clusters of short survival, the null and alternative hypotheses are written as:

$${H_0}:{\theta _{{\text{in}}}} = {\theta _{{\text{out}}}}\;{\text{for all}}\;z \in Z\;vs.\;{H_a}\;{\theta _{{\text{in}}}} < {\theta _{{\text{out}}}}\;{\text{for some}}\;z \in Z$$

where ${\theta }_{\mathrm{i}\mathrm{n}}$ denotes the mean survival time for subjects within zone $z$, and ${\theta }_{\mathrm{o}\mathrm{u}\mathrm{t}}$ is the mean survival time for subjects outside zone $z$. The exponential-based spatial scan statistic is defined as

$$\mathrm{\lambda }=\frac{\underset{z}{\mathrm{max}}{\left(\frac{{r}_{\mathrm{i}\mathrm{n}}}{\sum _{i\in z}{t}_{i}}\right)}^{{r}_{\mathrm{i}\mathrm{n}}}{\left(\frac{{r}_{\mathrm{o}\mathrm{u}\mathrm{t}}}{\sum _{i\notin z}{t}_{i}}\right)}^{{r}_{\mathrm{o}\mathrm{u}\mathrm{t}}}}{{\left(\frac{R}{\sum _{i\in G}{t}_{i}}\right)}^{R}}$$

where ${r}_{\mathrm{i}\mathrm{n}}=\sum _{i\in z}{\delta }_{i}$ and ${r}_{\mathrm{o}\mathrm{u}\mathrm{t}}=\sum _{i\notin z}{\delta }_{i}$ (the number of non-censored subjects inside and outside zone $z$, respectively). The total number of non-censored subjects in the entire study area $G$ is denoted by $R={r}_{\mathrm{i}\mathrm{n}}+{r}_{\mathrm{o}\mathrm{u}\mathrm{t}}.$When there are no censored observations, ${r}_{\mathrm{i}\mathrm{n}}$ and ${r}_{\mathrm{o}\mathrm{u}\mathrm{t}}$ are replaced by the total number of subjects inside and outside zone $z$, ${n}_{\mathrm{i}\mathrm{n}}$ and ${n}_{\mathrm{o}\mathrm{u}\mathrm{t}}$, respectively, with $R$ by $N={n}_{\mathrm{i}\mathrm{n}}+{n}_{\mathrm{o}\mathrm{u}\mathrm{t}}$ in the above test statistic.

When searching for clusters of short survival time using SaTScan™, users can specify the maximum size for *z*. The default setting is 50% of the total population. When the size of the most likely cluster is very large, one may want to know if smaller clusters that are statistically significant are contained in the large cluster. We can try different values for the maximum reported cluster size (MRCS), not the maximum scanning window size (MSWS). The MRCS is also set to 50% of the total population by default. It is not clear how to select the best MRCS for the exponential model. In the next section, we propose a Gini coefficient to optimize the MRCS for the exponential model.

### Gini coefficient for exponential model

To measure the disproportion of survival in each area, the Lorenz curve can be defined using the number of subjects and the sum of survival times. We define the x-axis as the cumulative proportion of the number of non-censored subjects and the y-axis as the cumulative proportion of the sum of observed times. If there is only one significant cluster ${z}^{*},$ the Lorenz curve is constructed in the same way as that of the Poisson model. Specifically, the x- and y-coordinates of point P for the cluster are defined as:

$${x}_{1}=\frac{\sum _{i\in {z}^{*}}{\delta }_{i}}{\sum _{i\in G}{\delta }_{i}}\left(=\frac{{r}_{\mathrm{i}\mathrm{n}}}{R}\right)$$

and

$${y}_{1}=\frac{\sum _{i\in {z}^{*}}{t}_{i}}{\sum _{i\in G}{t}_{i}}.$$

Considering the maximum likelihood estimates for the parameter $\theta$ of the exponential distribution under the null and alternative hypotheses, that is, ${\widehat{\theta }}_{0}=R/\sum _{i\in G}{t}_{i}$ and ${\widehat{\theta }}_{in}={r}_{in}/\sum _{i\in z}{t}_{i}$, the cumulative proportion of the sum of the observed times would be proportional to the cumulative proportion of non-censored subjects in each region under the null hypothesis of no clusters. If there is a significant cluster ${z}^{*}$ of short survival, the proportion of the sum of observed times in the cluster to that in the whole study region $G$ would be less than the proportion of the number of subjects. As the sum of the observed times in the cluster ${z^*}$ decreases, the y-coordinate ${y}_{1}$ decreases and the Lorenz curve moves farther away from the reference line. Then, the value of the Gini coefficient, which is twice the area between the Lorenz curve and the reference line, increases. When there are *K* clusters $z_1^*,\; \ldots ,\;z_K^*$ (ordered by their statistical significance), the coordinates of each cluster $({x}_{k},{y}_{k})$ are defined as ${x}_{k}=\sum _{i\in \left\{{\bigcup }_{j=1}^{k}{z}_{j}^{*}\right\}}{\delta }_{i}/R$ and ${y_k} = \mathop \sum \nolimits_{i \in \left\{{\bigcup }_{j=1}^{k} {z}_{j}^{*}\right\}} {t_i}/\mathop \sum \nolimits_{i \in G} {t_i}$. The Lorenz curve connects *K* points of $({x}_{k},{y}_{k})$, and the Gini coefficient is calculated in the same way as ${\sum }_{k=1}^{K+1}({y}_{k}{x}_{k-1}-{y}_{k-1}{x}_{k})$ with ${x}_{0}={y}_{0}=0$ and ${x}_{K+1}={y}_{K+1}=1.$ Different values for the MRCS produces different sets of clusters with different values of the Gini coefficient. We can select the optimal collection of clusters with the highest dissimilarity in survival based on the Gini coefficient.

### Simulation study

**Fig. 1:**
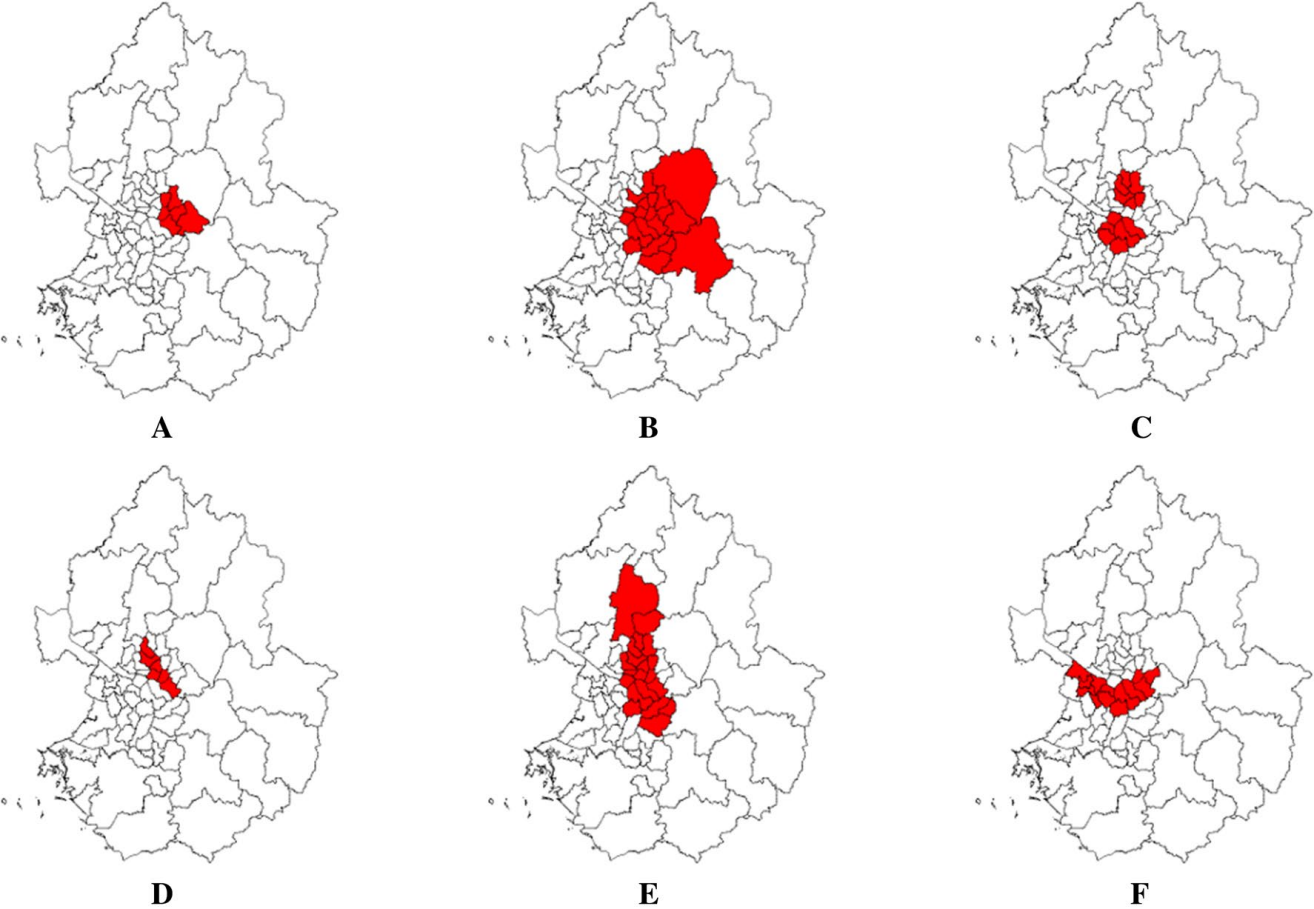
Cluster models used in the simulation. **A** one circular cluster of 10%, **B** one circular cluster of 30%, **C** two circular clusters of 10% each, **D** one elliptical cluster of 10%, **E** one elliptical cluster of 30%, **F** one irregular cluster of 20%

We conducted a simulation study to evaluate the performance of the Gini coefficient in the exponential model. We used six cluster models in Seoul and Gyeonggi Province in South Korea as the whole study region. True clusters of different shapes and sizes are assumed in the study region, consisting of 67 districts, as shown in Fig. 1. Since circular and elliptical windows are available in SaTScan™, we mainly considered these two shapes. We also included an irregularly shaped cluster to examine whether the proposed method could possibly work better in identifying irregular clusters than the default setting. Cluster models A and B assumed a circular true cluster of 10% (6 districts) and 30% (20 districts) of the entire study region, respectively. Cluster model C included two adjacent circular clusters, each of which accounts for 10% (6 districts). Models D and E consisted of elliptical clusters of 10% (6 districts) and 30% (20 districts). Model F included an irregularly shaped cluster of 20% (13 districts). For each model, we considered 12 scenarios for the combination of mean survival time and censoring rate. We varied the mean survival time for the true clusters as 2, 5, and 7, compared to 10 for areas outside the clusters. We adopted the parameter setting for the mean survival time from the study by Huang et al. [4]. The censoring rates were set to 10%, 30%, 50%, and 70% to examine how the performance of the proposed method can be affected by the censoring rate.

We generated 1,000 subjects and randomly assigned them to one of the 67 districts in the study region under each scenario. If a subject was in the districts of the true cluster, the survival time was generated from an exponential distribution with a mean of 2, 5, and 7. Otherwise, the survival time was generated from an exponential distribution with a mean of 10. We censored the survival time for randomly selected subjects out of the 1,000 subjects at a chosen censoring rate. We then searched for clusters with short survival using circular and elliptical scanning windows, with 15 MRCS values of 3%, 4%, 5%, 6%, 8%, 10%, 12%, 15%, 20%, 25%, 30%, 35%, 40%, 45%, and 50% in the SaTScan™ software. Using these numbers can be thought of as a grid search. These candidate MRCS values are used for the Poisson and Bernoulli models in SaTScan™ and were used for consistency with the exponential model. We selected these numbers to be consistent for the exponential model as used in the Poisson and Bernoulli models in SaTScan™. The MSWS was fixed at 50%. The Gini coefficient was calculated for each MRCS value. We selected the optimal MRCS with the highest Gini coefficient. The reported clusters were then compared with the true clusters.

We repeated the simulation 1,000 times for each scenario. We counted the number of times the Gini coefficient selected each of the 15 MRCS values as the optimal. The performance of the proposed method was summarized using the sensitivity and positive predicted value (PPV). In the context of spatial cluster detection, sensitivity is the proportion of districts correctly detected among the districts in the true cluster, and PPV is the proportion of districts correctly detected among the districts in the detected cluster. Higher values of these measures indicate more accurate detection. Specifically, the sensitivity and PPV were estimated from 1,000 datasets as

$${\text{Sensitivity}} = \frac{1}{S}\mathop \sum \limits_{s = 1}^S \frac{{number\;of\;districts\;correctly\;detected\;}}{{number\;of\;districts\;in\;the\;true\;cluster\;}}$$

$${\text{PPV}} = \frac{1}{S}\mathop \sum \limits_{s = 1}^S \frac{{number\;of\;districts\;correctly\;detected\;}}{{number\;of\;detected\;districts\;}}$$

where $S$ is the number of rejected datasets. We also calculated the accuracy measures under the default MRCS setting of 50% in SaTScan™.

### Korea community health survey data

To illustrate the proposed method, we used data from the Korea Community Health Survey (KCHS) conducted by the Korea Centers for Disease Control and Prevention [21]. This community-based cross-sectional survey has been conducted at 253 community health centres annually since 2008. The survey questionnaire includes topics related to health behaviour and prevention. We used the age of first drinking for males as the survival time in the 2017 survey data. If a person had never had a drink, his survival time was censored at the age of the survey. The location information of each individual was available at the district level because each district in Korea has approximately one community health centre. In Seoul and Gyeonggi province, we searched for areas with low mean age of first drinking (i.e. spatial clusters of short survival time) using the exponential-based spatial scan statistic with both circular and elliptical scanning windows. The reported clusters selected optimally by the proposed method were compared with those at the default setting in SaTScan™.

## Results

### Simulation study results

Here, we have presented only a subset of all the simulation results. The other results are included in Additional file 1. Tables 1 and 2 show that the Gini coefficient most often selected the optimal MRCS as the same as the size of the true cluster using circular or elliptical windows when the true cluster was circular with a mean survival time of 5, regardless of the censoring rate. The detection accuracy was very high for the most frequently chosen MRCS. Both the sensitivity and PPV were above 0.95, which is higher than those at the default setting in most cases. The difference in the detection accuracy between the most often chosen MRCS and the default setting was larger when the true cluster was smaller (10%). The difference in PPV was even more pronounced. When the true cluster was medium sized (30%), the PPV was higher in every case at the most often chosen MRCS, while the sensitivity was slightly higher or similar. These results indicate that the spatial scan statistic without optimizing the MRCS tends to report a larger cluster than the true cluster, especially when the true cluster is small. A lower PPV implies that the detected cluster is larger because the number of detected clusters is in the denominator when calculating the PPV. We also summarized the overall detection accuracy when using the Gini coefficient over all the chosen MRCSs. Still, the sensitivity and PPV were higher than or similar to those at the default setting.

**Table 1:** Simulation results for cluster model A (one circular cluster, 10% of total area) with a mean survival time of 5

	% of cens		Maximum reported cluster size (MRCS)																Default Setting
			3%	4%	5%	6%	8%	10%	12%	15%	20%	25%	30%	35%	40%	45%	50%	Overall	
Circular window	10%	Frequency	2	5	15	22	48	*657*	110	76	23	15	8	4	2	1	1		989
		Sensitivity	0.013	0.333	0.600	0.576	0.635	*0.972*	0.944	0.983	0.978	0.978	0.979	1.000	1.000	1.000	1.000	0.934	0.928
		PPV	0.500	1.000	0.931	0.957	0.885	*0.989*	0.814	0.670	0.503	0.428	0.322	0.286	0.235	0.222	0.188	0.909	0.903
	30%	Frequency	0	5	6	32	27	*540*	162	75	77	34	6	3	3	1	1		972
		Sensitivity	–	0.367	0.389	0.563	0.599	*0.957*	0.937	0.960	0.961	0.995	1.000	1.000	1.000	1.000	1	0.923	0.906
		PPV	–	0.950	0.911	0.974	0.783	*0.968*	0.812	0.700	0.524	0.430	0.339	0.271	0.234	0.214	0.188	0.852	0.845
	50%	Frequency	0	9	0	120	16	*367*	209	159	56	32	8	7	1	2	0		986
		Sensitivity	–	0.333	-	0.504	0.490	*0.972*	0.896	0.984	0.946	0.969	1.000	1.000	1.000	0.917	-	0.886	0.875
		PPV	–	1.000	-	0.991	0.716	*0.977*	0.774	0.711	0.479	0.419	0.327	0.301	0.231	0.165	-	0.830	0.824
	70%	Frequency	0	20	5	18	0	*531*	234	5	8	31	29	6	3	16	12		918
		Sensitivity	–	0.333	0.333	0.500	-	*0.970*	0.828	0.667	0.979	0.844	0.994	1.000	0.889	0.969	0.986	0.902	0.831
		PPV	–	1.000	1	0.986	-	*0.997*	0.708	0.500	0.522	0.349	0.319	0.269	0.219	0.208	0.191	0.841	0.837
Elliptical window	10%	Frequency	1	4	9	17	74	*430*	241	124	46	22	5	5	4	1	2		985
		Sensitivity	0.010	0.417	0.482	0.510	0.701	*0.957*	0.974	0.989	0.989	1.000	1.000	0.967	1.000	1.000	1.000	0.934	0.920
		PPV	1.000	1	0.963	1.000	0.962	*0.969*	0.823	0.689	0.522	0.399	0.326	0.279	0.238	0.207	0.185	0.853	0.847
	30%	Frequency	0	3	1	28	118	*248*	247	179	85	43	12	3	2	0	2		971
		Sensitivity	–	0.333	0.333	0.500	0.675	*0.940*	0.942	0.990	0.973	0.996	0.986	1.000	0.917	-	1.000	0.908	0.886
		PPV	–	1	1.000	0.988	0.968	*0.949*	0.799	0.690	0.538	0.411	0.331	0.287	0.220	-	0.164	0.794	0.788
	50%	Frequency	2	6	3	60	122	159	*258*	211	77	54	18	3	0	2	2		977
		Sensitivity	0.027	0.389	0.556	0.500	0.669	0.957	*0.913*	0.982	0.957	0.988	0.982	1.000	-	1.000	1.000	0.883	0.865
		PPV	1.000	1.000	0.933	0.992	0.986	0.955	*0.791*	0.696	0.522	0.411	0.321	0.288	-	0.182	0.185	0.781	0.779
	70%	Frequency	0	4	0	53	184	*212*	169	85	91	30	25	20	20	7	13		913
		Sensitivity	–	0.333	-	0.500	0.688	0.970	0.871	0.794	0.839	0.861	0.853	1.000	0.942	0.976	0.987	0.829	0.758
		PPV	–	1.000	-	1.000	0.987	0.986	0.770	0.535	0.460	0.340	0.275	0.280	0.227	0.207	0.182	0.762	0.761

% of cens, percentage of censoring; PPV, positive predictive value

Cells most often selected as the optimal MRCS are shown in italics

**Table 2:** Simulation results for cluster model B (one circular cluster, 30% of total area) with a mean survival time of 5

	% of cens		Maximum reported cluster size (MRCS)																Default Setting
			3%	4%	5%	6%	8%	10%	12%	15%	20%	25%	30%	35%	40%	45%	50%	Overall	
Circular window	10%	Frequency	0	0	0	0	0	1	1	1	1	22	*792*	166	16	0	0		1000
		Sensitivity	–	–	–	–	–	0.850	0.900	0.800	0.700	0.793	*0.985*	0.996	0.994	–	–	0.982	0.982
		PPV	–	–	–	–	–	0.895	0.783	1.000	0.667	1.000	*1.000*	0.928	0.820	–	–	0.984	0.985
	30%	Frequency	0	0	0	0	0	0	2	1	4	61	*678*	252	2	0	0		1000
		Sensitivity	–	–	–	–	–	–	0.725	0.700	0.513	0.76	*0.975*	0.999	0.925	–	–	0.966	0.966
		PPV	–	–	–	–	–	–	1.000	0.778	1.000	0.986	*0.999*	0.935	0.771	–	–	0.981	0.982
	50%	Frequency	0	0	0	0	0	0	3	0	4	42	*800*	149	2	0	0		1000
		Sensitivity	–	–	–	–	–	–	0.733	-	0.563	0.810	*0.976*	1.000	0.875	–	–	0.970	0.970
		PPV	–	–	–	–	–	–	1.000	-	1.000	0.998	*0.999*	0.924	0.716	–	–	0.987	0.987
	70%	Frequency	0	0	0	0	0	0	0	0	1	8	*851*	113	9	18	0		1000
		Sensitivity	–	–	–	–	–	–	–	–	0.450	0.744	*0.909*	0.989	0.978	0.989	–	0.918	0.918
		PPV	–	–	–	–	–	–	–	–	1.000	1.000	*0.997*	0.912	0.778	0.699	–	0.980	0.980
Elliptical window	10%	Frequency	0	0	0	0	0	0	1	4	3	24	*757*	176	31	3	1		1000
		Sensitivity	–	–	–	–	–	–	0.700	0.875	0.717	0.796	*0.981*	0.988	0.987	1.000	1.000	0.977	0.977
		PPV	–	–	–	–	–	–	0.875	0.825	0.955	0.995	*0.997*	0.921	0.796	0.732	0.606	0.975	0.976
	30%	Frequency	0	0	0	0	0	0	2	0	13	90	*619*	262	12	1	1		1000
		Sensitivity	–	–	–	–	–	–	0.750	-	0.627	0.761	*0.970*	0.992	0.983	1.000	1.000	0.952	0.953
		PPV	–	–	–	–	–	–	0.917	-	1.000	0.991	*0.997*	0.926	0.766	0.741	0.606	0.974	0.973
	50%	Frequency	0	0	0	0	0	0	1	0	6	74	*750*	156	10	2	1		1000
		Sensitivity	–	–	–	–	–	–	0.750	-	0.633	0.764	*0.967*	0.990	0.970	1.000	1.000	0.954	0.953
		PPV	–	–	–	–	–	–	1.000	-	1.000	0.995	*0.995*	0.916	0.765	0.742	0.625	0.980	0.979
	70%	Frequency	0	0	0	0	0	0	0	1	3	15	*666*	156	147	8	4		1000
		Sensitivity	–	–	–	–	–	–	–	0.900	0.583	0.753	*0.907*	0.956	0.993	0.969	1.000	0.925	0.925
		PPV	–	–	–	–	–	–	–	0.900	1.000	1.000	*0.997*	0.876	0.812	0.686	0.616	0.947	0.946

% of cens, percentage of censoring; PPV, positive predictive value

Cells most often selected as the optimal MRCS are shown in italics

In the case of two true clusters, which are close to each other, the proposed method often chose a slightly smaller MRCS than that of the true cluster. However, the PPV was always higher than that at the default setting, although the sensitivity was slightly lower only when the mean survival time in the true clusters was 5. This result again implied that the default setting reported rather a larger cluster than the true clusters. When the mean survival time was 7 in the true clusters, the frequency of chosen MRCS was spread over all possible MRCSs (Table 3). This might be attributable to the low detection power due to the small difference in mean survival time inside vs. outside the clusters. The promising indication here is that the overall sensitivity is much higher when using the Gini coefficient than without it.

**Table 3:** Simulation results for cluster model C (two circular clusters, 10% each of total area) with a mean survival time of 7

	% of cens		Maximum reported cluster size (MRCS)																Default Setting
			3%	4%	5%	6%	8%	10%	12%	15%	20%	25%	30%	35%	40%	45%	50%	Overall	
Circular window	10%	Frequency	18	12	11	17	*103*	48	46	65	19	12	20	39	53	39	26		528
		Sensitivity	0.005	0.174	0.288	0.284	*0.414*	0.464	0.478	0.544	0.513	0.597	0.771	0.857	0.943	0.953	0.971	0.588	0.313
		PPV	0.982	0.875	0.947	0.931	*0.971*	0.819	0.724	0.711	0.547	0.484	0.495	0.468	0.451	0.393	0.361	0.694	0.690
	30%	Frequency	30	7	15	15	*114*	46	55	82	28	34	17	26	18	23	17		527
		Sensitivity	0.007	0.167	0.244	0.256	*0.382*	0.409	0.444	0.498	0.506	0.674	0.750	0.840	0.917	0.971	0.980	0.500	0.267
		PPV	0.967	1.000	0.778	0.967	*0.986*	0.799	0.736	0.724	0.525	0.525	0.485	0.455	0.439	0.393	0.360	0.734	0.734
	50%	Frequency	47	4	17	8	*82*	24	44	58	26	49	11	23	27	28	17		465
		Sensitivity	0.011	0.167	0.245	0.250	*0.383*	0.438	0.451	0.471	0.458	0.692	0.735	0.844	0.874	0.911	0.927	0.505	0.242
		PPV	1.000	1.000	0.878	0.833	*0.937*	0.824	0.733	0.687	0.487	0.535	0.485	0.453	0.431	0.381	0.343	0.697	0.692
	70%	Frequency	77	2	15	3	*281*	46	5	14	7	10	23	27	3	53	25		591
		Sensitivity	0.016	0.167	0.250	0.250	*0.356*	0.379	0.383	0.399	0.500	0.658	0.757	0.904	0.778	0.849	0.827	0.424	0.258
		PPV	0.987	1.000	0.750	0.600	*0.764*	0.697	0.582	0.506	0.482	0.492	0.486	0.477	0.378	0.357	0.321	0.691	0.691
Elliptical window	10%	Frequency	4	14	14	17	*73*	43	54	39	48	55	55	39	39	24	33		551
		Sensitivity	0.001	0.196	0.268	0.304	*0.389*	0.446	0.435	0.532	0.547	0.811	0.861	0.949	0.949	0.951	0.975	0.636	0.350
		PPV	1.000	0.905	0.869	0.985	*0.908*	0.826	0.709	0.692	0.574	0.623	0.573	0.530	0.460	0.388	0.354	0.664	0.662
	30%	Frequency	14	23	6	22	84	*86*	52	64	43	50	34	20	22	25	15		560
		Sensitivity	0.004	0.217	0.236	0.299	0.367	*0.420*	0.431	0.475	0.574	0.777	0.851	0.900	0.905	0.960	0.983	0.539	0.304
		PPV	1.000	1.000	0.944	0.967	0.936	*0.871*	0.751	0.666	0.581	0.609	0.548	0.491	0.439	0.400	0.359	0.729	0.729
	50%	Frequency	27	26	9	20	54	*59*	39	78	46	50	23	22	20	25	33		531
		Sensitivity	0.007	0.244	0.222	0.300	0.360	*0.412*	0.436	0.473	0.533	0.717	0.710	0.845	0.900	0.937	0.927	0.526	0.285
		PPV	1.000	1.000	0.861	0.905	0.887	*0.802*	0.740	0.647	0.542	0.570	0.440	0.465	0.437	0.389	0.339	0.673	0.670
	70%	Frequency	34	23	2	54	*154*	122	17	15	35	14	32	37	40	20	49		648
		Sensitivity	0.010	0.221	0.208	0.323	*0.329*	0.336	0.343	0.383	0.521	0.637	0.779	0.887	0.892	0.883	0.959	0.481	0.316
		PPV	1.000	1.000	0.708	0.973	*0.762*	0.594	0.529	0.498	0.536	0.478	0.492	0.484	0.432	0.372	0.357	0.646	0.645

% of cens, percentage of censoring; PPV, positive predictive value

Cells most often selected as the optimal MRCS are shown in italics

In the case of elliptical clusters, the Gini coefficient with elliptical scanning windows most often picked the best MRCS of the same size as the true cluster when the mean survival time was 5 inside the true cluster (Tables 4 and 5). When the cluster was small (10%), the detection accuracy at the most often chosen MRCS was much higher than that at the default setting. When the mean survival time was 2 inside the true cluster, similar patterns were observed. The Gini coefficient with circular scanning windows most often selected a smaller MRCS than the true cluster size. Still, the overall sensitivity and PPV at the most often chosen MRCS were higher than those at the default setting. When the mean survival time was 7 inside the true cluster, the overall detection accuracy was higher than that at the default setting.

**Table 4:** Simulation results for cluster model D (one elliptical cluster, 10% of total area) with a mean survival time of 5

	% of cens		Maximum reported cluster size (MRCS)																Default Setting
			3%	4%	5%	6%	8%	10%	12%	15%	20%	25%	30%	35%	40%	45%	50%	Overall	
Circular window	10%	Frequency	14	3	8	196	*256*	82	20	41	84	57	36	17	20	6	5		845
		Sensitivity	0.017	0.278	0.500	0.669	*0.650*	0.689	0.700	0.797	0.863	0.974	0.986	0.971	0.992	1.000	1.000	0.730	0.617
		PPV	1.000	0.778	0.800	0.970	*0.891*	0.697	0.553	0.509	0.461	0.410	0.328	0.270	0.239	0.200	0.172	0.728	0.726
	30%	Frequency	6	3	10	240	*365*	92	7	35	63	44	11	11	4	5	2		898
		Sensitivity	0.011	0.333	0.617	0.653	*0.655*	0.714	0.714	0.805	0.833	0.920	0.985	1.000	0.958	0.967	1.000	0.699	0.629
		PPV	0.833	1.000	0.860	0.989	*0.910*	0.678	0.568	0.501	0.453	0.385	0.327	0.270	0.212	0.192	0.182	0.807	0.802
	50%	Frequency	3	0	8	87	*415*	49	7	37	90	84	9	16	16	7	19		847
		Sensitivity	0.003	–	0.667	0.634	*0.659*	0.660	0.619	0.766	0.782	0.865	0.926	0.979	0.990	0.929	0.974	0.716	0.612
		PPV	0.667	–	0.767	0.966	*0.867*	0.694	0.531	0.503	0.425	0.353	0.296	0.270	0.227	0.196	0.176	0.698	0.691
	70%	Frequency	0	0	2	25	*142*	9	1	7	25	32	99	32	5	77	40		496
		Sensitivity	-	–	0.500	0.640	*0.612*	0.648	0.333	0.762	0.807	0.823	0.978	0.995	0.967	0.903	0.883	0.807	0.426
		PPV	-	–	1.000	0.960	*0.768*	0.707	0.286	0.491	0.444	0.325	0.329	0.265	0.228	0.191	0.171	0.464	0.437
Elliptical window	10%	Frequency	1	5	14	28	67	*426*	140	101	64	34	15	11	8	4	2		920
		Sensitivity	0.004	0.300	0.488	0.667	0.704	*0.974*	0.973	0.965	0.961	0.976	0.978	0.985	0.958	1.000	1.000	0.931	0.857
		PPV	1.000	0.800	0.976	0.966	0.945	*0.953*	0.817	0.657	0.525	0.404	0.327	0.278	0.229	0.205	0.191	0.820	0.820
	30%	Frequency	2	2	9	57	68	*460*	160	96	52	17	8	3	3	2	2		941
		Sensitivity	0.006	0.333	0.556	0.670	0.696	*0.959*	0.959	0.953	0.910	0.951	0.958	0.944	0.944	0.917	1.000	0.912	0.858
		PPV	0.500	1.000	0.944	0.994	0.973	*0.937*	0.819	0.663	0.493	0.405	0.305	0.257	0.226	0.174	0.188	0.847	0.848
	50%	Frequency	0	3	6	3	54	*340*	248	134	54	37	11	6	12	4	12		924
		Sensitivity	–	0.222	0.472	0.722	0.735	*0.960*	0.962	0.969	0.880	0.896	0.864	0.972	0.958	0.958	0.972	0.934	0.864
		PPV	–	0.667	0.944	0.875	0.944	*0.939*	0.825	0.674	0.473	0.369	0.279	0.265	0.230	0.187	0.182	0.785	0.783
	70%	Frequency	0	1	12	13	105	*301*	18	38	21	41	39	30	60	20	34		733
		Sensitivity	–	0.333	0.472	0.500	0.730	*0.976*	0.759	0.886	0.746	0.850	0.897	0.956	0.986	0.942	0.966	0.893	0.657
		PPV	–	1.000	0.944	1.000	0.981	*0.924*	0.667	0.594	0.416	0.349	0.290	0.265	0.241	0.204	0.182	0.692	0.690

% of cens, percentage of censoring; PPV, positive predictive value

Cells most often selected as the optimal MRCS are shown in italics

**Table 5:** Simulation results for cluster model E (one elliptical cluster, 30% of total area) with a mean survival time of 5

	% of cens		Maximum reported cluster size (MRCS)																Default Setting
			3%	4%	5%	6%	8%	10%	12%	15%	20%	25%	30%	35%	40%	45%	50%	Overall	
Circular window	10%	Frequency	0	0	0	0	3	24	92	128	62	44	20	*334*	249	15	29		1000
		Sensitivity	–	–	–	–	0.500	0.669	0.709	0.790	0.764	0.755	0.830	*0.885*	0.896	0.890	0.962	0.841	0.810
		PPV	–	–	–	–	0.926	0.997	0.994	0.978	0.890	0.849	0.781	*0.776*	0.757	0.639	0.588	0.826	0.820
	30%	Frequency	0	0	0	1	8	18	155	209	83	91	20	*257*	144	5	9		1000
		Sensitivity	–	–	–	0.600	0.556	0.536	0.694	0.736	0.692	0.665	0.760	*0.882*	0.896	0.890	0.933	0.778	0.725
		PPV	–	–	–	0.923	0.983	1.000	0.992	0.973	0.918	0.853	0.765	*0.775*	0.768	0.675	0.577	0.872	0.863
	50%	Frequency	0	0	0	0	1	22	69	124	71	94	16	*395*	193	3	12		1000
		Sensitivity	–	–	–	–	0.300	0.627	0.612	0.665	0.563	0.632	0.747	*0.880*	0.900	0.900	0.913	0.785	0.765
		PPV	–	–	–	–	1.000	1.000	0.992	0.970	0.960	0.854	0.809	*0.778*	0.768	0.645	0.557	0.837	0.836
	70%	Frequency	3	0	0	0	4	6	8	18	46	45	15	*680*	32	92	51		1000
		Sensitivity	0.067	–	–	–	0.200	0.425	0.506	0.697	0.551	0.672	0.770	*0.878*	0.892	0.872	0.913	0.840	0.836
		PPV	1.000	–	–	–	1.000	1.000	0.935	0.941	0.958	0.815	0.758	*0.765*	0.704	0.624	0.553	0.758	0.758
Elliptical window	10%	Frequency	0	0	0	0	0	2	11	52	42	72	*428*	273	101	18	1		1000
		Sensitivity	–	–	–	–	–	0.625	0.696	0.867	0.755	0.765	*0.894*	0.951	0.959	0.992	1.000	0.899	0.896
		PPV	–	–	–	–	–	0.962	0.966	0.930	0.931	0.993	*0.980*	0.891	0.795	0.709	0.606	0.928	0.933
	30%	Frequency	0	0	0	0	1	1	22	49	63	137	*543*	138	35	9	2		1000
		Sensitivity	–	–	–	–	0.700	0.550	0.700	0.816	0.652	0.720	*0.893*	0.936	0.954	0.978	1.000	0.854	0.853
		PPV	–	–	–	–	1.000	1.000	0.976	0.927	0.962	0.980	*0.983*	0.881	0.791	0.680	0.616	0.954	0.956
	50%	Frequency	0	0	0	0	0	3	1	41	68	121	*512*	170	60	21	3		1000
		Sensitivity	–	–	–	–	–	0.617	0.700	0.762	0.624	0.707	*0.894*	0.931	0.950	0.983	1.000	0.858	0.858
		PPV	–	–	–	–	–	1.000	0.933	0.942	0.981	0.966	*0.985*	0.856	0.788	0.698	0.619	0.940	0.943
	70%	Frequency	0	0	0	0	1	1	3	10	19	49	*371*	126	260	85	75		1000
		Sensitivity	–	–	–	–	0.200	0.650	0.633	0.625	0.629	0.722	*0.873*	0.901	0.952	0.977	0.995	0.899	0.899
		PPV	–	–	–	–	1.000	1.000	0.921	0.882	0.996	0.921	*0.963*	0.801	0.755	0.693	0.617	0.837	0.838

% of cens, percentage of censoring; PPV, positive predictive value

Cells most often selected as the optimal MRCS are shown in italics

When the true cluster was irregularly shaped, the proposed method seemed to choose smaller sizes of MRCS than the true cluster size. However, the overall sensitivity was always higher than that at the default setting. When the mean survival time was 7 in the true cluster, the difference in performance was clearer (Table 6). This might be because refined sets of smaller clusters were reported by the Gini coefficient rather than a single larger cluster.

**Table 6:** Simulation results for cluster model F (one irregular cluster, 20% of total area) with a mean survival time of 7

	% of cens		Maximum reported cluster size (MRCS)																Default Setting
			3%	4%	5%	6%	8%	10%	12%	15%	20%	25%	30%	35%	40%	45%	50%	Overall	
Circular window	10%	Frequency	20	6	13	27	*69*	37	57	38	33	45	17	20	37	32	45		496
		Sensitivity	0.005	0.141	0.266	0.279	*0.360*	0.383	0.418	0.490	0.543	0.656	0.697	0.792	0.963	0.966	0.978	0.563	0.285
		PPV	0.950	0.750	0.930	0.951	*0.966*	0.788	0.719	0.673	0.567	0.568	0.460	0.459	0.481	0.434	0.394	0.674	0.663
	30%	Frequency	20	2	15	12	53	21	*70*	33	49	55	11	17	43	19	40		460
		Sensitivity	0.004	0.154	0.226	0.282	0.373	0.374	*0.409*	0.501	0.584	0.641	0.769	0.810	0.964	0.931	0.983	0.575	0.267
		PPV	0.950	1.000	0.878	0.979	0.973	0.785	*0.775*	0.678	0.582	0.557	0.511	0.482	0.484	0.411	0.398	0.670	0.663
	50%	Frequency	41	1	36	27	31	11	*135*	30	50	39	3	15	59	15	26		519
		Sensitivity	0.008	0.154	0.220	0.271	0.360	0.385	*0.404*	0.474	0.500	0.602	0.667	0.821	0.979	0.903	0.950	0.498	0.263
		PPV	1.000	1.000	0.940	0.938	0.967	0.755	*0.754*	0.637	0.557	0.534	0.453	0.488	0.491	0.411	0.399	0.700	0.698
	70%	Frequency	66	0	16	4	*204*	30	106	23	24	37	1	1	10	31	109		662
		Sensitivity	0.015	–	0.226	0.308	*0.331*	0.382	0.411	0.452	0.583	0.605	0.615	0.769	0.923	0.849	0.924	0.473	0.317
		PPV	0.985	–	0.797	0.764	*0.787*	0.736	0.743	0.607	0.622	0.496	0.421	0.455	0.468	0.394	0.399	0.681	0.681
Elliptical window	10%	Frequency	4	6	14	16	31	49	60	71	*104*	80	39	49	41	29	26		619
		Sensitivity	0.001	0.154	0.247	0.313	0.347	0.424	0.512	0.574	*0.673*	0.779	0.862	0.906	0.934	0.942	0.965	0.668	0.414
		PPV	1.000	0.833	0.929	0.972	0.966	0.899	0.879	0.816	*0.751*	0.659	0.591	0.521	0.477	0.421	0.383	0.716	0.714
	30%	Frequency	9	5	5	9	14	34	62	72	*79*	50	63	60	42	24	33		561
		Sensitivity	0.002	0.231	0.231	0.325	0.352	0.459	0.529	0.585	*0.631*	0.800	0.865	0.919	0.951	0.952	0.974	0.704	0.396
		PPV	0.944	1.000	0.933	1.000	0.954	0.951	0.932	0.835	*0.716*	0.666	0.594	0.538	0.498	0.431	0.392	0.705	0.703
	50%	Frequency	30	12	10	8	22	26	65	74	*93*	89	69	50	23	18	24		613
		Sensitivity	0.009	0.224	0.223	0.317	0.332	0.396	0.501	0.577	*0.663*	0.792	0.845	0.906	0.953	0.932	0.968	0.650	0.402
		PPV	1.000	1.000	0.933	0.888	0.899	0.883	0.904	0.813	*0.758*	0.667	0.578	0.541	0.490	0.426	0.395	0.726	0.727
	70%	Frequency	12	6	5	15	84	210	29	100	*247*	32	34	17	8	29	18		846
		Sensitivity	0.007	0.205	0.277	0.303	0.362	0.458	0.480	0.600	*0.685*	0.762	0.697	0.810	0.817	0.918	0.932	0.578	0.490
		PPV	1.000	1.000	1.000	0.911	0.926	0.856	0.846	0.869	*0.810*	0.654	0.495	0.484	0.434	0.422	0.395	0.797	0.797

% of cens, percentage of censoring; PPV, positive predictive value

Cells most often selected as the optimal MRCS are shown in italics

### KCHS data analysis results

**Fig. 2:**
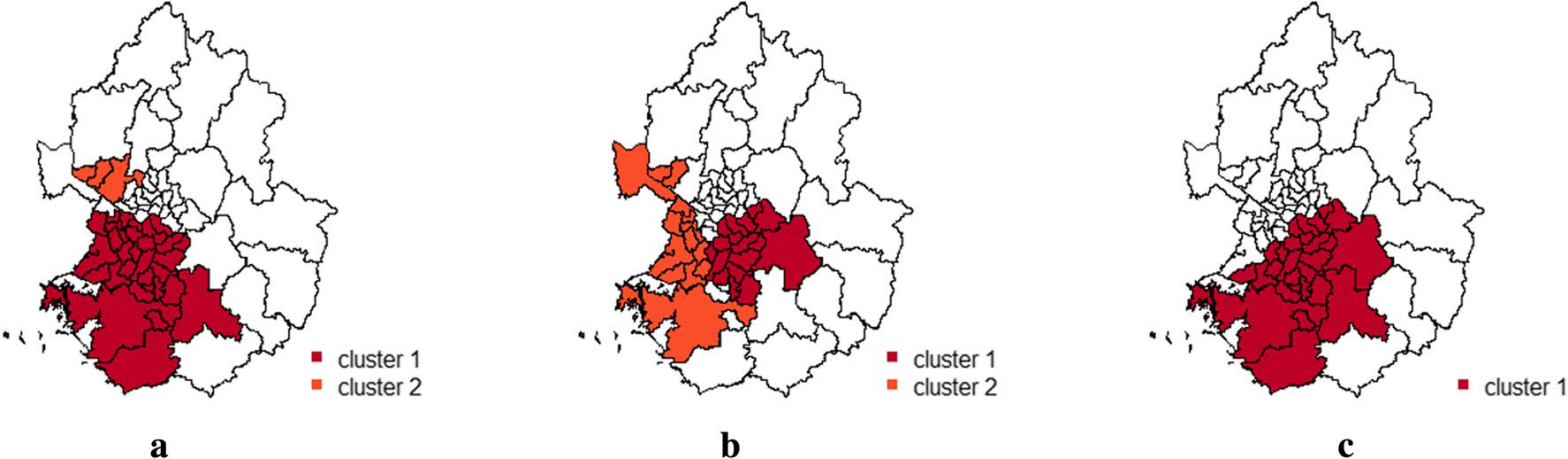
Spatial clusters with low mean age of first drinking in Seoul and Gyeonggi province using 2017 KCHS data. **a** circular windows, Gini or default (50%), **b** elliptical windows, Gini (25%), **c** elliptical windows, default (50%)

When using circular windows, the proposed method selected the default setting of 50% as the optimal MRCS. The most likely cluster was quite large, including 31 districts, as shown in Fig. 2(a). A small secondary cluster consisting of three districts was also detected. When using elliptical windows, the proposed method selected 25% as the optimal MRCS. The detected clusters were slightly different from those at the default setting. Information on the detected clusters is presented in Table 7. A single large cluster consisting of 26 districts was detected at the default setting (Fig. 2(c)), while two smaller clusters were detected using the Gini coefficients (Fig. 2(b)). Cluster 1 in Fig. 2(b) is part of cluster 1 in Fig. 2(c). Some districts of cluster 2 in Fig. 2(b) overlapped with cluster 1 in Fig. 2(c), but the other districts were not included in the cluster in Fig. 2(c). The test statistic value for the cluster in Fig. 2(c) was much larger than that for cluster 1 in Fig. 2(b). However, the mean survival time of cluster 1 in Fig. 2(b) was lower than that of cluster 1 in Fig. 2(c). It is likely that the default setting detected a larger cluster by including unnecessary neighbouring districts. Although the mean survival time of cluster 2 in Fig. 2(b) was higher than that of cluster 1 in Fig. 2(c), it was still lower than that outside the clusters and is statistically significant. The clusters at the optimal MRCS chosen by the Gini coefficient in Fig. 2(b) appear to be more meaningful than cluster 1 in Fig. 2(c).

**Table 7:** Cluster detection results for 2017 KCHS data using elliptical windows with the Gini coefficient and default setting for MRCS

	Cluster	Districts^a^	LLR	p-value	Mean survival time	Observations^a^	Non-censored
Gini (25%)	1	16	26.73	0.001	21.34	6584	6313
	2	15	9.88	0.001	22.10	7073	6706
Default	1	26	47.12	0.001	21.51	11,271	10,781

^a^Districts- number of districts; *LLR* log-likelihood ratio; ^a^Observations- number of observations; ^a^Non-censored- number of non-censored observations

## Discussion and conclusion

We have proposed the Gini coefficient in the exponential-based spatial scan statistic to optimize the MRCS in cluster detection analysis for survival data. The proposed method was defined to measure the degree of heterogeneity in the mean survival times of clusters. Our simulation study showed that the Gini coefficient mostly selected the optimal MRCS, similar to the true cluster size. The detection accuracy was higher for the best chosen MRCS than at the default setting. A lower PPV at the default setting indicates that using the default value of 50% of the total population for the MSWS and MRCS tends to produce a larger cluster that hides smaller clusters and includes non-informative areas. Even though the Gini coefficient did not always select the optimal MRCS the same as the true cluster size, the overall detection accuracy when using the Gini coefficient was generally improved compared to when it was not used. This improvement was greatly noticeable in some cases.

The application of the proposed method to the KCHS data supported that the proposed method can optimize the MRCS in spatial cluster detection analysis for survival data. We searched for a cluster with a short survival time. The most likely cluster at the default setting was rather larger with a higher mean survival time than that at the optimal MRCS chosen by the Gini coefficient. Interestingly, the two clusters at the optimal MRCS were contiguous and formed an irregularly shaped cluster. As reported by Kim and Jung [12], the Gini coefficient might also be useful for detecting irregularly shaped clusters in the exponential model.

Here, we again emphasize that we optimize the MRCS using the Gini coefficient, not the MSWS. Rerunning the analyses with different MSWSs should be avoided because of the multiple testing problem. Wang et al. [22] presented their proposed method, called the maximum clustering heterogeneous set proportion, as an indicator to select the MSWS. As they described, different MSWSs lead to different sets of windows and then different detected clusters. Thus, even the same cluster under different sets of windows can have different p-values. It is incorrect to choose the result with the smallest p-value because it is not appropriately adjusted for multiple testing. Trying different values of MRCS to select clusters for reporting is the correct way to do this.

The Gini coefficient was first developed for the Poisson and Bernoulli models and subsequently adopted for the ordinal and normal-based models. The Gini coefficient for the exponential model in this study was also specifically defined for the specific probability model and thoroughly evaluated. The option to optimize the MRCS using the Gini coefficient in SaTScan™ is available only for the Poisson and Bernoulli models. It is easy to implement the Gini coefficient in the exponential model using R with the ‘rsatscan’ package[23]. An R function to calculate the Gini coefficient is available upon request.

Using the spatial scan statistic with the default setting has been criticized because the detected most likely cluster may be much larger than the true clusters as they might include irrelevant neighbouring areas [24–27]. Studies that proposed the Gini coefficient for the Poisson, Bernoulli, ordinal, and normal models revealed that using the Gini coefficient in spatial scan statistics can resolve this problem to a certain extent [11, 13, 14]. Using the Gini coefficient for the Poisson model can also be effective in detecting irregularly shaped clusters [12]. The exponential model can be used for spatial cluster detection analysis of time-to-event type data such as cancer survival, time to disease recurrence, or age at first smoking, with or without censoring. We believe that using the Gini coefficient in the exponential-based spatial scan statistic can be very helpful for reporting more refined and informative clusters for survival data.

## Supplementary Information

**Addtional file 1: Table A1.** Simulation results for cluster model A (one circular cluster, 10% of total area) with a mean survival time of 2. **Table A2.** Simulation results for cluster model A (one circular cluster, 10% of total area) with a mean survival time of 7. **Table A3.** Simulation results for cluster model B (one circular cluster, 30% of total area) with a mean survival time of 2. **Table A4.** Simulation results for cluster model B (one circular cluster, 30% of total area) with a mean survival time of 7. **Table A5.** Simulation results for cluster model C (two circular clusters, 10% each of total area) with a mean survival time of 2. **Table A6.** Simulation results for cluster model C (two circular clusters, 10% each of total area) with a mean survival time of 5. **Table A7.** Simulation results for cluster model D (one elliptic cluster, 10% of total area) with a mean survival time of 2. **Table A8.** Simulation results for cluster model D (one elliptic cluster, 10% of total area) with a mean survival time of 7. **Table A9.** Simulation results for cluster model E (one elliptic cluster, 30% of total area) with a mean survival time of 2. **Table A10.** Simulation results for cluster model E (one elliptic cluster, 30% of total area) with a mean survival time of 7.

## Data Availability

The datasets used and/or analysed during the current study are available from the corresponding author on reasonable request.

## Abbreviations

MRCS: Maximum reported cluster size
MSWS: Maximum scanning window size
KCHS: Korea Community Health Survey
