# Supplementary material for: Optimizing the maximum reported cluster size in the spatial scan statistic for survival data
Source: Int J Health Geogr. 2021 Jul 8;20:33. doi: 10.1186/s12942-021-00286-w (PMC8265152; doi:10.1186/s12942-021-00286-w)
Supplement: Supplementary file 1 — Addtional file 1: Table A1. Simulation results for cluster model A (one circular cluster, 10% of total area) with a mean survival time of 2. Table A2. Simulation results for cluster model A (one circular cluster, 10% of total area) with a mean survival time of 7. Table A3. Simulation results for cluster model B (one circular cluster, 30% of total area) with a mean survival time of 2. Table A4. Simulation results for cluster model B (one circular cluster, 30% of total area) with a mean survival time of 7. Table A5. Simulation results for cluster model C (two circular clusters, 10% each of total area) with a mean survival time of 2. Table A6. Simulation results for cluster model C (two circular clusters, 10% each of total area) with a mean survival time of 5. Table A7. Simulation results for cluster model D (one elliptic cluster, 10% of total area) with a mean survival time of 2. Table A8. Simulation results for cluster model D (one elliptic cluster, 10% of total area) with a mean survival time of 7. Table A9. Simulation results for cluster model E (one elliptic cluster, 30% of total area) with a mean survival time of 2. Table A10. Simulation results for cluster model E (one elliptic cluster, 30% of total area) with a mean survival time of 7. [file 12942_2021_286_MOESM1_ESM.docx]

Table A1. Simulation results for cluster model A (one circular cluster, 10% of total area) with a mean survival time of 2

|  | | | Maximum reported cluster size (MRCS) | | | | | | | | | | | | | | | | Default Setting |
| --- | --- | --- | --- | --- | --- | --- | --- | --- | --- | --- | --- | --- | --- | --- | --- | --- | --- | --- | --- |
|  | % of cens |  | 3% | 4% | 5% | 6% | 8% | 10% | 12% | 15% | 20% | 25% | 30% | 35% | 40% | 45% | 50% | Overall |  |
| Circular window | 10% | Frequency | 13 | 29 | 52 | 42 | 6 | 858 | 0 | 0 | 0 | 0 | 0 | 0 | 0 | 0 | 0 |  | 1000 |
|  |  | Sensitivity | 1.000 | 0.999 | 0.937 | 0.989 | 0.833 | 1.000 | - | - | - | - | - | - | - | - | - | 0.995 | 0.997 |
|  |  | PPV | 0.995 | 0.997 | 0.927 | 0.851 | 0.759 | 1.000 | - | - | - | - | - | - | - | - | - | 0.988 | 0.973 |
|  | 30% | Frequency | 23 | 93 | 67 | 80 | 16 | 720 | 1 | 0 | 0 | 0 | 0 | 0 | 0 | 0 | 0 |  | 1000 |
|  |  | Sensitivity | 1.000 | 1.000 | 0.992 | 0.997 | 0.865 | 1.000 | 1.000 | - | - | - | - | - | - | - | - | 0.997 | 0.996 |
|  |  | PPV | 0.997 | 0.986 | 0.976 | 0.858 | 0.738 | 1.000 | 0.857 | - | - | - | - | - | - | - | - | 0.981 | 0.956 |
|  | 50% | Frequency | 0 | 1 | 39 | 139 | 13 | 794 | 10 | 4 | 0 | 0 | 0 | 0 | 0 | 0 | 0 |  | 1000 |
|  |  | Sensitivity | - | 0.8333 | 0.8846 | 0.988 | 0.8974 | 0.9985 | 1 | 1 | - | - | - | - | - | - | - | 0.9911 | 0.9977 |
|  |  | PPV | - | 0.8333 | 0.8309 | 0.855 | 0.7531 | 0.9977 | 0.8571 | 0.75 | - | - | - | - | - | - | - | 0.9656 | 0.9946 |
|  | 70% | Frequency | 0 | 0 | 0 | 133 | 1 | 864 | 2 | 0 | 0 | 0 | 0 | 0 | 0 | 0 | 0 |  | 1000 |
|  |  | Sensitivity | - | - | - | 1.000 | 1.000 | 0.997 | 1.000 | - | - | - | - | - | - | - | - | 0.998 | 0.998 |
|  |  | PPV | - | - | - | 0.856 | 0.462 | 0.998 | 0.857 | - | - | - | - | - | - | - | - | 0.978 | 0.998 |
| Elliptic window | 10% | Frequency | 2 | 10 | 43 | 88 | 12 | 833 | 12 | 0 | 0 | 0 | 0 | 0 | 0 | 0 | 0 |  | 1000 |
|  |  | Sensitivity | 1.000 | 0.950 | 0.841 | 0.905 | 1.000 | 0.999 | 1.000 | - | - | - | - | - | - | - | - | 0.983 | 0.999 |
|  |  | PPV | 0.875 | 0.814 | 0.841 | 0.724 | 0.747 | 1.000 | 0.857 | - | - | - | - | - | - | - | - | 0.962 | 0.998 |
|  | 30% | Frequency | 1 | 6 | 57 | 56 | 6 | 845 | 29 | 0 | 0 | 0 | 0 | 0 | 0 | 0 | 0 |  | 1000 |
|  |  | Sensitivity | 0.833 | 0.944 | 0.877 | 0.923 | 1.000 | 0.999 | 1.000 | - | - | - | - | - | - | - | - | 0.988 | 0.999 |
|  |  | PPV | 1.000 | 0.857 | 0.877 | 0.769 | 0.733 | 1.000 | 0.850 | - | - | - | - | - | - | - | - | 0.973 | 0.995 |
|  | 50% | Frequency | 2 | 7 | 55 | 73 | 3 | 810 | 44 | 5 | 1 | 0 | 0 | 0 | 0 | 0 | 0 |  | 1000 |
|  |  | Sensitivity | 0.833 | 0.857 | 0.952 | 0.938 | 0.667 | 1.000 | 1.000 | 1.000 | 1.000 | - | - | - | - | - | - | 0.990 | 0.997 |
|  |  | PPV | 1.000 | 0.878 | 0.952 | 0.820 | 1.000 | 0.999 | 0.857 | 0.733 | 0.600 | - | - | - | - | - | - | 0.975 | 0.991 |
|  | 70% | Frequency | 0 | 4 | 0 | 0 | 4 | 988 | 3 | 0 | 1 | 0 | 0 | 0 | 0 | 0 | 0 |  | 1000 |
|  |  | Sensitivity | - | 0.833 | - | - | 0.833 | 0.998 | 1.000 | - | 1.000 | - | - | - | - | - | - | 0.996 | 0.996 |
|  |  | PPV | - | 0.958 | - | - | 0.875 | 0.999 | 0.857 | - | 0.600 | - | - | - | - | - | - | 0.997 | 0.999 |

% of cens, percentage of censoring; PPV, positive predictive value.

Cells most often selected as the optimal MRCS are shaded in grey.

Table A2. Simulation results for cluster model A (one circular cluster, 10% of total area) with a mean survival time of 7

|  | | | Maximum reported cluster size (MRCS) | | | | | | | | | | | | | | | | Default Setting |
| --- | --- | --- | --- | --- | --- | --- | --- | --- | --- | --- | --- | --- | --- | --- | --- | --- | --- | --- | --- |
|  | % of cens |  | 3% | 4% | 5% | 6% | 8% | 10% | 12% | 15% | 20% | 25% | 30% | 35% | 40% | 45% | 50% | Overall |  |
| Circular window | 10% | Frequency | 9 | 6 | 14 | 17 | 36 | 69 | 32 | 44 | 40 | 14 | 12 | 9 | 10 | 10 | 7 |  | 329 |
|  |  | Sensitivity | 0.002 | 0.194 | 0.393 | 0.441 | 0.579 | 0.896 | 0.828 | 0.939 | 0.858 | 0.952 | 0.944 | 1.000 | 1.000 | 0.967 | 0.952 | 0.787 | 0.281 |
|  |  | PPV | 0.500 | 0.583 | 0.833 | 0.873 | 0.784 | 0.923 | 0.712 | 0.656 | 0.448 | 0.386 | 0.313 | 0.282 | 0.243 | 0.205 | 0.177 | 0.649 | 0.599 |
|  | 30% | Frequency | 8 | 3 | 7 | 11 | 18 | 42 | 40 | 26 | 46 | 19 | 8 | 9 | 3 | 3 | 5 |  | 248 |
|  |  | Sensitivity | 0.000 | 0.111 | 0.381 | 0.439 | 0.509 | 0.794 | 0.688 | 0.776 | 0.913 | 0.930 | 0.979 | 0.889 | 0.944 | 0.944 | 0.933 | 0.741 | 0.207 |
|  |  | PPV | 0.250 | 0.333 | 1.000 | 0.841 | 0.701 | 0.800 | 0.602 | 0.538 | 0.484 | 0.390 | 0.323 | 0.249 | 0.233 | 0.195 | 0.175 | 0.566 | 0.516 |
|  | 50% | Frequency | 7 | 6 | 3 | 65 | 18 | 23 | 76 | 52 | 79 | 33 | 8 | 13 | 5 | 5 | 9 |  | 402 |
|  |  | Sensitivity | 0.001 | 0.278 | 0.278 | 0.495 | 0.509 | 0.870 | 0.704 | 0.894 | 0.793 | 0.950 | 1.000 | 1.000 | 0.967 | 0.967 | 0.944 | 0.739 | 0.314 |
|  |  | PPV | 0.429 | 0.833 | 0.667 | 0.978 | 0.708 | 0.870 | 0.608 | 0.625 | 0.413 | 0.408 | 0.337 | 0.296 | 0.221 | 0.179 | 0.171 | 0.600 | 0.572 |
|  | 70% | Frequency | 0 | 4 | 2 | 5 | 5 | 16 | 69 | 3 | 7 | 23 | 21 | 6 | 4 | 30 | 26 |  | 221 |
|  |  | Sensitivity | - | 0.250 | 0.333 | 0.100 | 0.067 | 0.802 | 0.703 | 0.667 | 0.786 | 0.761 | 0.992 | 1.000 | 0.917 | 0.928 | 0.949 | 0.778 | 0.179 |
|  |  | PPV | - | 0.750 | 1.000 | 0.200 | 0.100 | 0.839 | 0.602 | 0.500 | 0.426 | 0.310 | 0.318 | 0.274 | 0.224 | 0.196 | 0.182 | 0.420 | 0.392 |
| Elliptic window | 10% | Frequency | 3 | 8 | 12 | 13 | 33 | 39 | 48 | 58 | 50 | 28 | 13 | 15 | 13 | 5 | 7 |  | 345 |
|  |  | Sensitivity | 0.001 | 0.250 | 0.431 | 0.449 | 0.581 | 0.774 | 0.816 | 0.874 | 0.923 | 0.964 | 0.974 | 0.978 | 0.987 | 0.967 | 0.952 | 0.803 | 0.291 |
|  |  | PPV | 0.667 | 0.708 | 0.889 | 0.865 | 0.786 | 0.786 | 0.697 | 0.600 | 0.483 | 0.379 | 0.312 | 0.275 | 0.233 | 0.193 | 0.176 | 0.587 | 0.556 |
|  | 30% | Frequency | 2 | 10 | 8 | 20 | 44 | 40 | 70 | 70 | 98 | 82 | 28 | 28 | 12 | 8 | 6 |  | 526 |
|  |  | Sensitivity | 0.000 | 0.233 | 0.417 | 0.500 | 0.636 | 0.700 | 0.714 | 0.833 | 0.867 | 0.951 | 0.893 | 0.929 | 0.944 | 0.958 | 1.000 | 0.797 | 0.218 |
|  |  | PPV | 1.000 | 0.700 | 0.917 | 0.975 | 0.841 | 0.734 | 0.630 | 0.567 | 0.471 | 0.384 | 0.293 | 0.265 | 0.229 | 0.204 | 0.184 | 0.541 | 0.521 |
|  | 50% | Frequency | 8 | 8 | 22 | 56 | 56 | 106 | 132 | 124 | 142 | 100 | 44 | 36 | 10 | 24 | 8 |  | 876 |
|  |  | Sensitivity | 0.001 | 0.167 | 0.333 | 0.494 | 0.566 | 0.591 | 0.707 | 0.817 | 0.845 | 0.917 | 0.909 | 0.907 | 1.000 | 0.972 | 1.000 | 0.743 | 0.335 |
|  |  | PPV | 0.750 | 0.500 | 0.667 | 0.938 | 0.798 | 0.637 | 0.629 | 0.565 | 0.459 | 0.379 | 0.303 | 0.251 | 0.238 | 0.196 | 0.185 | 0.544 | 0.532 |
|  | 70% | Frequency | 0 | 3 | 2 | 4 | 13 | 6 | 28 | 30 | 47 | 23 | 27 | 15 | 21 | 11 | 28 |  | 258 |
|  |  | Sensitivity | - | 0.111 | 0.333 | 0.375 | 0.539 | 0.528 | 0.685 | 0.700 | 0.706 | 0.746 | 0.728 | 1.000 | 0.929 | 0.970 | 0.982 | 0.758 | 0.196 |
|  |  | PPV | - | 0.333 | 0.833 | 0.750 | 0.739 | 0.600 | 0.625 | 0.488 | 0.379 | 0.293 | 0.237 | 0.274 | 0.222 | 0.207 | 0.183 | 0.380 | 0.375 |

% of cens, percentage of censoring; PPV, positive predictive value.

Table A3. Simulation results for cluster model B (one circular cluster, 30% of total area) with a mean survival time of 2

|  | | | Maximum reported cluster size (MRCS) | | | | | | | | | | | | | | | | Default Setting |
| --- | --- | --- | --- | --- | --- | --- | --- | --- | --- | --- | --- | --- | --- | --- | --- | --- | --- | --- | --- |
|  | % of cens |  | 3% | 4% | 5% | 6% | 8% | 10% | 12% | 15% | 20% | 25% | 30% | 35% | 40% | 45% | 50% | Overall |  |
| Circular window | 10% | Frequency | 0 | 0 | 0 | 0 | 0 | 2 | 0 | 47 | 1 | 0 | 949 | 1 | 0 | 0 | 0 |  | 1000 |
|  |  | Sensitivity | - | - | - | - | - | 0.975 | - | 0.850 | 0.850 | - | 0.999 | 1.000 | - | - | - | 0.992 | 0.999 |
|  |  | PPV | - | - | - | - | - | 0.907 | - | 0.810 | 0.850 | - | 1.000 | 0.952 | - | - | - | 0.991 | 1.000 |
|  | 30% | Frequency | 0 | 0 | 0 | 0 | 4 | 0 | 1 | 19 | 0 | 1 | 971 | 4 | 0 | 0 | 0 |  | 1000 |
|  |  | Sensitivity | - | - | - | - | 0.900 | - | 0.900 | 0.847 | - | 0.900 | 1.000 | 1.000 | - | - | - | 0.996 | 1.000 |
|  |  | PPV | - | - | - | - | 0.900 | - | 0.783 | 0.809 | - | 0.900 | 1.000 | 0.952 | - | - | - | 0.996 | 1.000 |
|  | 50% | Frequency | 0 | 0 | 0 | 0 | 1 | 1 | 0 | 5 | 1 | 0 | 992 | 0 | 0 | 0 | 0 |  | 1000 |
|  |  | Sensitivity | - | - | - | - | 0.900 | 0.950 | - | 0.850 | 0.850 | - | 1.000 | - | - | - | - | 0.999 | 1.000 |
|  |  | PPV | - | - | - | - | 0.900 | 0.905 | - | 0.810 | 0.895 | - | 1.000 | - | - | - | - | 0.999 | 1.000 |
|  | 70% | Frequency | 0 | 0 | 0 | 0 | 0 | 0 | 0 | 136 | 0 | 0 | 862 | 2 | 0 | 0 | 0 |  | 1000 |
|  |  | Sensitivity | - | - | - | - | - | - | - | 0.750 | - | - | 0.992 | 1.000 | - | - | - | 0.959 | 0.991 |
|  |  | PPV | - | - | - | - | - | - | - | 0.790 | - | - | 1.000 | 0.952 | - | - | - | 0.971 | 1.000 |
| Elliptic window | 10% | Frequency | 0 | 0 | 0 | 0 | 0 | 1 | 7 | 0 | 0 | 0 | 991 | 1 | 0 | 0 | 0 |  | 1000 |
|  |  | Sensitivity | - | - | - | - | - | 1.000 | 0.957 | - | - | - | 0.999 | 1.000 | - | - | - | 0.999 | 0.999 |
|  |  | PPV | - | - | - | - | - | 0.667 | 0.667 | - | - | - | 1.000 | 0.952 | - | - | - | 0.997 | 1.000 |
|  | 30% | Frequency | 0 | 0 | 0 | 0 | 0 | 1 | 8 | 4 | 0 | 0 | 983 | 4 | 0 | 0 | 0 |  | 1000 |
|  |  | Sensitivity | - | - | - | - | - | 1.000 | 0.950 | 0.800 | - | - | 1.000 | 1.000 | - | - | - | 0.998 | 1.000 |
|  |  | PPV | - | - | - | - | - | 0.606 | 0.658 | 0.820 | - | - | 1.000 | 0.952 | - | - | - | 0.996 | 1.000 |
|  | 50% | Frequency | 0 | 0 | 0 | 0 | 0 | 0 | 5 | 0 | 0 | 0 | 995 | 0 | 0 | 0 | 0 |  | 1000 |
|  |  | Sensitivity | - | - | - | - | - | - | 0.950 | - | - | - | 1.000 | - | - | - | - | 1.000 | 0.999 |
|  |  | PPV | - | - | - | - | - | - | 0.704 | - | - | - | 1.000 | - | - | - | - | 0.999 | 1.000 |
|  | 70% | Frequency | 0 | 0 | 0 | 0 | 0 | 0 | 0 | 5 | 0 | 0 | 993 | 2 | 0 | 0 | 0 |  | 1000 |
|  |  | Sensitivity | - | - | - | - | - | - | - | 0.750 | - | - | 0.990 | 1.000 | - | - | - | 0.989 | 0.991 |
|  |  | PPV | - | - | - | - | - | - | - | 0.790 | - | - | 1.000 | 0.952 | - | - | - | 0.999 | 1.000 |

% of cens, percentage of censoring; PPV, positive predictive value.

Cells most often selected as the optimal MRCS are shaded in grey.

Table A4. Simulation results for cluster model B (one circular cluster, 30% of total area) with a mean survival time of 7

|  | | | Maximum reported cluster size (MRCS) | | | | | | | | | | | | | | | | Default Setting |
| --- | --- | --- | --- | --- | --- | --- | --- | --- | --- | --- | --- | --- | --- | --- | --- | --- | --- | --- | --- |
|  | % of cens |  | 3% | 4% | 5% | 6% | 8% | 10% | 12% | 15% | 20% | 25% | 30% | 35% | 40% | 45% | 50% | Overall |  |
| Circular window | 10% | Frequency | 2 | 1 | 1 | 1 | 8 | 2 | 13 | 14 | 31 | 115 | 351 | 212 | 80 | 30 | 39 |  | 900 |
|  |  | Sensitivity | 0.001 | 0.150 | 0.150 | 0.150 | 0.225 | 0.325 | 0.339 | 0.421 | 0.511 | 0.739 | 0.927 | 0.959 | 0.959 | 0.922 | 0.951 | 0.871 | 0.787 |
|  |  | PPV | 1.000 | 1.000 | 1.000 | 1.000 | 0.969 | 1.000 | 0.951 | 0.974 | 0.943 | 0.969 | 0.976 | 0.882 | 0.772 | 0.648 | 0.581 | 0.905 | 0.891 |
|  | 30% | Frequency | 10 | 1 | 1 | 0 | 5 | 2 | 18 | 22 | 88 | 232 | 232 | 222 | 20 | 13 | 33 |  | 899 |
|  |  | Sensitivity | 0.007 | 0.100 | 0.150 | - | 0.230 | 0.300 | 0.339 | 0.430 | 0.540 | 0.717 | 0.955 | 0.914 | 0.908 | 0.919 | 0.950 | 0.798 | 0.722 |
|  |  | PPV | 1.000 | 0.667 | 1.000 | - | 1.000 | 1.000 | 1.000 | 0.985 | 0.976 | 0.960 | 0.881 | 0.973 | 0.716 | 0.629 | 0.574 | 0.922 | 0.904 |
|  | 50% | Frequency | 8 | 0 | 0 | 2 | 3 | 3 | 13 | 11 | 157 | 164 | 350 | 174 | 27 | 26 | 25 |  | 963 |
|  |  | Sensitivity | 0.013 | - | - | 0.175 | 0.217 | 0.300 | 0.346 | 0.414 | 0.523 | 0.727 | 0.923 | 0.961 | 0.870 | 0.940 | 0.944 | 0.804 | 0.776 |
|  |  | PPV | 1.000 | - | - | 1.000 | 1.000 | 1.000 | 1.000 | 0.990 | 0.983 | 0.974 | 0.978 | 0.883 | 0.677 | 0.649 | 0.556 | 0.933 | 0.921 |
|  | 70% | Frequency | 21 | 0 | 0 | 2 | 0 | 0 | 23 | 5 | 26 | 68 | 378 | 136 | 20 | 172 | 63 |  | 914 |
|  |  | Sensitivity | 0.013 | - | - | 0.100 | - | - | 0.346 | 0.400 | 0.517 | 0.640 | 0.847 | 0.915 | 0.920 | 0.944 | 0.887 | 0.819 | 0.750 |
|  |  | PPV | 1.000 | - | - | 0.400 | - | - | 1.000 | 1.000 | 0.952 | 0.883 | 0.955 | 0.824 | 0.738 | 0.661 | 0.538 | 0.843 | 0.841 |
| Elliptic window | 10% | Frequency | 2 | 1 | 1 | 3 | 7 | 9 | 11 | 12 | 39 | 107 | 254 | 213 | 137 | 59 | 47 |  | 902 |
|  |  | Sensitivity | 0.001 | 0.100 | 0.150 | 0.183 | 0.207 | 0.328 | 0.336 | 0.408 | 0.537 | 0.736 | 0.886 | 0.932 | 0.959 | 0.968 | 0.960 | 0.854 | 0.775 |
|  |  | PPV | 1.000 | 0.667 | 1.000 | 0.917 | 0.900 | 1.000 | 0.941 | 0.933 | 0.946 | 0.956 | 0.941 | 0.856 | 0.770 | 0.686 | 0.592 | 0.862 | 0.849 |
|  | 30% | Frequency | 5 | 3 | 0 | 5 | 9 | 9 | 16 | 26 | 113 | 175 | 193 | 185 | 100 | 38 | 29 |  | 906 |
|  |  | Sensitivity | 0.003 | 0.100 | - | 0.230 | 0.239 | 0.272 | 0.347 | 0.440 | 0.566 | 0.707 | 0.845 | 0.927 | 0.927 | 0.937 | 0.922 | 0.773 | 0.707 |
|  |  | PPV | 1.000 | 0.667 | - | 1.000 | 1.000 | 0.926 | 0.986 | 0.991 | 0.972 | 0.949 | 0.921 | 0.854 | 0.738 | 0.659 | 0.565 | 0.881 | 0.863 |
|  | 50% | Frequency | 4 | 3 | 1 | 3 | 5 | 10 | 16 | 22 | 108 | 196 | 285 | 167 | 68 | 43 | 27 |  | 958 |
|  |  | Sensitivity | 0.007 | 0.100 | 0.150 | 0.250 | 0.220 | 0.295 | 0.350 | 0.414 | 0.538 | 0.712 | 0.860 | 0.922 | 0.939 | 0.957 | 0.939 | 0.780 | 0.751 |
|  |  | PPV | 1.000 | 0.667 | 1.000 | 1.000 | 1.000 | 1.000 | 1.000 | 0.959 | 0.973 | 0.967 | 0.940 | 0.848 | 0.742 | 0.673 | 0.568 | 0.899 | 0.890 |
|  | 70% | Frequency | 11 | 4 | 0 | 0 | 3 | 1 | 11 | 6 | 31 | 45 | 221 | 197 | 167 | 107 | 125 |  | 929 |
|  |  | Sensitivity | 0.008 | 0.100 | - | - | 0.183 | 0.250 | 0.318 | 0.367 | 0.523 | 0.660 | 0.935 | 0.900 | 0.811 | 0.921 | 0.964 | 0.852 | 0.795 |
|  |  | PPV | 1.000 | 0.667 | - | - | 0.733 | 1.000 | 0.886 | 0.866 | 0.938 | 0.886 | 0.754 | 0.816 | 0.910 | 0.660 | 0.596 | 0.781 | 0.774 |

% of cens, percentage of censoring; PPV, positive predictive value.

Cells most often selected as the optimal MRCS are shaded in grey.

Table A5. Simulation results for cluster model C (two circular clusters, 10% each of total area) with a mean survival time of 2

|  | | | Maximum reported cluster size (MRCS) | | | | | | | | | | | | | | | | Default Setting |
| --- | --- | --- | --- | --- | --- | --- | --- | --- | --- | --- | --- | --- | --- | --- | --- | --- | --- | --- | --- |
|  | % of cens |  | 3% | 4% | 5% | 6% | 8% | 10% | 12% | 15% | 20% | 25% | 30% | 35% | 40% | 45% | 50% | Overall |  |
| Circular window | 10% | Frequency | 0 | 20 | 163 | 136 | 545 | 77 | 54 | 3 | 0 | 0 | 0 | 0 | 1 | 1 | 0 |  | 1000 |
|  |  | Sensitivity | - | 0.838 | 0.915 | 0.852 | 0.890 | 0.951 | 0.949 | 0.972 | - | - | - | - | 1.000 | 1.000 | - | 0.896 | 0.847 |
|  |  | PPV | - | 0.919 | 0.951 | 0.908 | 0.998 | 0.919 | 0.895 | 0.854 | - | - | - | - | 0.462 | 0.429 | - | 0.963 | 0.985 |
|  | 30% | Frequency | 0 | 13 | 142 | 190 | 500 | 40 | 104 | 9 | 0 | 0 | 0 | 0 | 1 | 0 | 1 |  | 1000 |
|  |  | Sensitivity | - | 0.833 | 0.909 | 0.822 | 0.876 | 0.956 | 0.922 | 0.945 | - | - | - | - | 1.000 | - | 1.000 | 0.879 | 0.846 |
|  |  | PPV | - | 0.909 | 0.934 | 0.908 | 0.995 | 0.918 | 0.860 | 0.832 | - | - | - | - | 0.522 | - | 0.571 | 0.949 | 0.977 |
|  | 50% | Frequency | 0 | 0 | 55 | 75 | 727 | 39 | 81 | 6 | 1 | 0 | 0 | 1 | 0 | 8 | 7 |  | 1000 |
|  |  | Sensitivity | - | - | 0.876 | 0.819 | 0.854 | 0.912 | 0.920 | 0.917 | 1.000 | - | - | 0.917 | - | 0.896 | 0.917 | 0.861 | 0.840 |
|  |  | PPV | - | - | 0.917 | 0.906 | 0.997 | 0.890 | 0.896 | 0.836 | 0.667 | - | - | 0.500 | - | 0.774 | 0.573 | 0.967 | 0.980 |
|  | 70% | Frequency | 0 | 0 | 14 | 6 | 821 | 56 | 34 | 9 | 0 | 0 | 2 | 0 | 40 | 7 | 11 |  | 1000 |
|  |  | Sensitivity | - | - | 0.893 | 0.750 | 0.821 | 0.887 | 0.917 | 0.917 | - | - | 0.792 | - | 0.852 | 0.893 | 0.902 | 0.832 | 0.822 |
|  |  | PPV | - | - | 0.915 | 0.938 | 0.991 | 0.889 | 0.846 | 0.786 | - | - | 0.903 | - | 0.825 | 0.825 | 0.711 | 0.966 | 0.969 |
| Elliptic window | 10% | Frequency | 0 | 6 | 132 | 20 | 411 | 332 | 2 | 1 | 5 | 60 | 28 | 3 | 0 | 0 | 0 |  | 1000 |
|  |  | Sensitivity | - | 0.903 | 0.968 | 0.938 | 0.914 | 0.934 | 0.917 | 1.000 | 0.750 | 0.954 | 0.988 | 1.000 | - | - | - | 0.932 | 0.922 |
|  |  | PPV | - | 0.903 | 0.967 | 0.957 | 1.000 | 0.982 | 0.917 | 0.706 | 0.750 | 0.696 | 0.681 | 0.734 | - | - | - | 0.959 | 0.965 |
|  | 30% | Frequency | 0 | 2 | 91 | 8 | 378 | 363 | 6 | 0 | 23 | 58 | 40 | 23 | 3 | 4 | 1 |  | 1000 |
|  |  | Sensitivity | - | 0.833 | 0.963 | 0.844 | 0.917 | 0.919 | 0.903 | - | 0.797 | 0.935 | 0.942 | 0.928 | 0.889 | 0.917 | 0.917 | 0.921 | 0.841 |
|  |  | PPV | - | 0.955 | 0.963 | 0.940 | 1.000 | 0.990 | 0.881 | - | 0.754 | 0.748 | 0.717 | 0.715 | 0.635 | 0.759 | 0.846 | 0.951 | 0.979 |
|  | 50% | Frequency | 0 | 1 | 39 | 0 | 396 | 319 | 28 | 4 | 53 | 76 | 29 | 41 | 12 | 1 | 1 |  | 1000 |
|  |  | Sensitivity | - | 0.917 | 0.938 | - | 0.914 | 0.917 | 0.952 | 0.958 | 0.921 | 0.932 | 0.940 | 0.927 | 0.924 | 0.917 | 0.917 | 0.920 | 0.778 |
|  |  | PPV | - | 0.917 | 0.951 | - | 1.000 | 0.991 | 0.862 | 0.823 | 0.831 | 0.792 | 0.731 | 0.742 | 0.743 | 0.647 | 0.786 | 0.944 | 0.981 |
|  | 70% | Frequency | 0 | 0 | 1 | 2 | 739 | 5 | 4 | 4 | 50 | 108 | 50 | 31 | 5 | 1 | 0 |  | 1000 |
|  |  | Sensitivity | - | - | 0.750 | 0.583 | 0.901 | 0.900 | 0.938 | 0.896 | 0.877 | 0.911 | 0.910 | 0.914 | 0.900 | 0.917 | - | 0.901 | 0.783 |
|  |  | PPV | - | - | 1.000 | 1.000 | 1.000 | 0.915 | 0.887 | 0.979 | 0.897 | 0.872 | 0.704 | 0.646 | 0.415 | 0.733 | - | 0.951 | 0.985 |

% of cens, percentage of censoring; PPV, positive predictive value.

Cells most often selected as the optimal MRCS are shaded in grey.

Table A6. Simulation results for cluster model C (two circular clusters, 10% each of total area) with a mean survival time of 5

|  | | | Maximum reported cluster size (MRCS) | | | | | | | | | | | | | | | | Default Setting |
| --- | --- | --- | --- | --- | --- | --- | --- | --- | --- | --- | --- | --- | --- | --- | --- | --- | --- | --- | --- |
|  | % of cens |  | 3% | 4% | 5% | 6% | 8% | 10% | 12% | 15% | 20% | 25% | 30% | 35% | 40% | 45% | 50% | Overall |  |
| Circular window | 10% | Frequency | 1 | 2 | 11 | 22 | 454 | 116 | 105 | 75 | 6 | 7 | 45 | 46 | 75 | 28 | 5 |  | 998 |
|  |  | Sensitivity | 0.028 | 0.167 | 0.303 | 0.303 | 0.404 | 0.478 | 0.492 | 0.500 | 0.528 | 0.655 | 0.850 | 0.944 | 0.987 | 0.982 | 0.967 | 0.535 | 0.534 |
|  |  | PPV | 1.000 | 1.000 | 0.970 | 1.000 | 0.996 | 0.846 | 0.807 | 0.731 | 0.552 | 0.538 | 0.531 | 0.503 | 0.475 | 0.410 | 0.370 | 0.831 | 0.831 |
|  | 30% | Frequency | 5 | 2 | 8 | 7 | 515 | 73 | 103 | 135 | 6 | 40 | 22 | 29 | 27 | 19 | 8 |  | 999 |
|  |  | Sensitivity | 0.097 | 0.167 | 0.240 | 0.262 | 0.405 | 0.435 | 0.484 | 0.500 | 0.542 | 0.733 | 0.826 | 0.897 | 0.997 | 0.991 | 0.990 | 0.493 | 0.493 |
|  |  | PPV | 1.000 | 1.000 | 0.865 | 1.000 | 1.000 | 0.834 | 0.812 | 0.743 | 0.566 | 0.559 | 0.525 | 0.479 | 0.488 | 0.412 | 0.364 | 0.857 | 0.856 |
|  | 50% | Frequency | 14 | 1 | 9 | 8 | 421 | 46 | 145 | 140 | 8 | 68 | 15 | 38 | 40 | 26 | 15 |  | 994 |
|  |  | Sensitivity | 0.100 | 0.167 | 0.241 | 0.250 | 0.412 | 0.455 | 0.498 | 0.497 | 0.500 | 0.733 | 0.767 | 0.906 | 0.973 | 0.971 | 0.994 | 0.524 | 0.521 |
|  |  | PPV | 1.000 | 1.000 | 0.907 | 0.906 | 0.998 | 0.839 | 0.837 | 0.735 | 0.554 | 0.558 | 0.505 | 0.480 | 0.476 | 0.411 | 0.373 | 0.822 | 0.822 |
|  | 70% | Frequency | 39 | 1 | 4 | 1 | 729 | 64 | 4 | 6 | 3 | 20 | 46 | 46 | 4 | 24 | 4 |  | 995 |
|  |  | Sensitivity | 0.089 | 0.167 | 0.250 | 0.250 | 0.395 | 0.412 | 0.438 | 0.417 | 0.472 | 0.721 | 0.752 | 0.937 | 1.000 | 0.934 | 0.792 | 0.449 | 0.448 |
|  |  | PPV | 1.000 | 1.000 | 0.750 | 0.600 | 0.923 | 0.776 | 0.656 | 0.528 | 0.470 | 0.544 | 0.493 | 0.489 | 0.480 | 0.392 | 0.314 | 0.846 | 0.848 |
| Elliptic window | 10% | Frequency | 2 | 0 | 5 | 15 | 157 | 201 | 69 | 49 | 29 | 171 | 184 | 69 | 31 | 10 | 6 |  | 998 |
|  |  | Sensitivity | 0.125 | - | 0.450 | 0.483 | 0.685 | 0.799 | 0.823 | 0.813 | 0.776 | 0.932 | 0.974 | 0.981 | 0.987 | 0.992 | 1.000 | 0.851 | 0.809 |
|  |  | PPV | 1.000 | - | 0.971 | 1.000 | 0.987 | 0.945 | 0.830 | 0.758 | 0.697 | 0.685 | 0.662 | 0.565 | 0.487 | 0.421 | 0.348 | 0.782 | 0.784 |
|  | 30% | Frequency | 1 | 6 | 4 | 43 | 221 | 290 | 69 | 64 | 57 | 112 | 72 | 27 | 19 | 6 | 6 |  | 997 |
|  |  | Sensitivity | 0.021 | 0.320 | 0.354 | 0.368 | 0.457 | 0.519 | 0.545 | 0.599 | 0.730 | 0.897 | 0.953 | 0.975 | 0.996 | 1.000 | 1.000 | 0.616 | 0.588 |
|  |  | PPV | 1.000 | 1.000 | 0.958 | 0.990 | 0.990 | 0.964 | 0.811 | 0.744 | 0.694 | 0.668 | 0.631 | 0.546 | 0.485 | 0.429 | 0.355 | 0.846 | 0.854 |
|  | 50% | Frequency | 4 | 19 | 11 | 44 | 191 | 289 | 64 | 81 | 41 | 112 | 61 | 35 | 19 | 11 | 13 |  | 995 |
|  |  | Sensitivity | 0.083 | 0.246 | 0.296 | 0.364 | 0.420 | 0.488 | 0.557 | 0.572 | 0.681 | 0.826 | 0.939 | 0.960 | 0.978 | 0.992 | 0.994 | 0.584 | 0.571 |
|  |  | PPV | 1.000 | 1.000 | 1.000 | 0.992 | 0.987 | 0.957 | 0.820 | 0.723 | 0.677 | 0.636 | 0.612 | 0.540 | 0.474 | 0.419 | 0.364 | 0.832 | 0.835 |
|  | 70% | Frequency | 8 | 14 | 3 | 212 | 340 | 98 | 17 | 7 | 50 | 38 | 88 | 70 | 32 | 5 | 12 |  | 994 |
|  |  | Sensitivity | 0.054 | 0.208 | 0.389 | 0.349 | 0.386 | 0.384 | 0.539 | 0.452 | 0.667 | 0.873 | 0.944 | 0.917 | 0.914 | 0.983 | 0.972 | 0.522 | 0.510 |
|  |  | PPV | 1.000 | 1.000 | 0.917 | 1.000 | 0.895 | 0.668 | 0.634 | 0.583 | 0.635 | 0.622 | 0.571 | 0.502 | 0.440 | 0.404 | 0.359 | 0.787 | 0.786 |

% of cens, percentage of censoring; PPV, positive predictive value.

Cells most often selected as the optimal MRCS are shaded in grey.

Table A7. Simulation results for cluster model D (one elliptic cluster, 10% of total area) with a mean survival time of 2

|  | | | Maximum reported cluster size (MRCS) | | | | | | | | | | | | | | | | Default Setting |
| --- | --- | --- | --- | --- | --- | --- | --- | --- | --- | --- | --- | --- | --- | --- | --- | --- | --- | --- | --- |
|  | % of cens |  | 3% | 4% | 5% | 6% | 8% | 10% | 12% | 15% | 20% | 25% | 30% | 35% | 40% | 45% | 50% | Overall |  |
| Circular window | 10% | Frequency | 48 | 8 | 119 | 511 | 233 | 30 | 25 | 9 | 8 | 5 | 2 | 0 | 0 | 1 | 1 |  | 1000 |
|  |  | Sensitivity | 0.948 | 0.896 | 0.910 | 0.946 | 0.951 | 0.994 | 1.000 | 1.000 | 1.000 | 1.000 | 1.000 | - | - | 0.833 | 1.000 | 0.946 | 0.838 |
|  |  | PPV | 0.992 | 0.815 | 0.838 | 0.931 | 0.900 | 0.697 | 0.640 | 0.540 | 0.600 | 0.527 | 0.504 | - | - | 0.278 | 0.462 | 0.890 | 0.891 |
|  | 30% | Frequency | 34 | 1 | 148 | 504 | 206 | 37 | 46 | 9 | 9 | 4 | 2 | 0 | 0 | 0 | 0 |  | 1000 |
|  |  | Sensitivity | 0.971 | 0.833 | 0.851 | 0.800 | 0.887 | 0.987 | 1.000 | 1.000 | 1.000 | 1.000 | 1.000 | - | - | - | - | 0.852 | 0.757 |
|  |  | PPV | 1.000 | 0.833 | 0.855 | 0.984 | 0.921 | 0.641 | 0.694 | 0.550 | 0.605 | 0.530 | 0.504 | - | - | - | - | 0.916 | 0.919 |
|  | 50% | Frequency | 62 | 2 | 210 | 469 | 226 | 15 | 8 | 1 | 4 | 0 | 1 | 0 | 0 | 1 | 1 |  | 1000 |
|  |  | Sensitivity | 0.737 | 0.667 | 0.848 | 0.824 | 0.871 | 0.889 | 1.000 | 0.833 | 0.917 | - | 1.000 | - | - | 1.000 | 1.000 | 0.837 | 0.765 |
|  |  | PPV | 0.997 | 0.733 | 0.833 | 0.990 | 0.906 | 0.670 | 0.724 | 0.556 | 0.602 | - | 0.316 | - | - | 0.600 | 0.194 | 0.927 | 0.932 |
|  | 70% | Frequency | 1 | 0 | 0 | 864 | 121 | 9 | 0 | 0 | 1 | 0 | 3 | 1 | 0 | 0 | 0 |  | 1000 |
|  |  | Sensitivity | 0.667 | - | - | 0.790 | 0.689 | 0.870 | - | - | 0.833 | - | 1.000 | 1.000 | - | - | - | 0.779 | 0.701 |
|  |  | PPV | 1.000 | - | - | 0.992 | 0.795 | 0.723 | - | - | 0.455 | - | 0.334 | 0.261 | - | - | - | 0.962 | 0.919 |
| Elliptic window | 10% | Frequency | 6 | 5 | 13 | 245 | 122 | 565 | 44 | 0 | 0 | 0 | 0 | 0 | 0 | 0 | 0 |  | 1000 |
|  |  | Sensitivity | 0.972 | 0.967 | 0.987 | 0.963 | 0.999 | 1.000 | 1.000 | - | - | - | - | - | - | - | - | 0.990 | 1.000 |
|  |  | PPV | 0.881 | 0.829 | 0.804 | 0.818 | 0.800 | 0.992 | 0.850 | - | - | - | - | - | - | - | - | 0.916 | 0.986 |
|  | 30% | Frequency | 10 | 10 | 9 | 158 | 98 | 680 | 35 | 0 | 0 | 0 | 0 | 0 | 0 | 0 | 0 |  | 1000 |
|  |  | Sensitivity | 0.983 | 0.900 | 0.926 | 0.908 | 0.998 | 1.000 | 1.000 | - | - | - | - | - | - | - | - | 0.984 | 0.999 |
|  |  | PPV | 0.871 | 0.851 | 0.870 | 0.797 | 0.804 | 0.987 | 0.857 | - | - | - | - | - | - | - | - | 0.931 | 0.985 |
|  | 50% | Frequency | 8 | 7 | 1 | 146 | 24 | 703 | 111 | 0 | 0 | 0 | 0 | 0 | 0 | 0 | 0 |  | 1000 |
|  |  | Sensitivity | 0.979 | 0.881 | 0.833 | 0.935 | 0.993 | 1.000 | 1.000 | - | - | - | - | - | - | - | - | 0.989 | 0.999 |
|  |  | PPV | 0.875 | 0.825 | 0.833 | 0.825 | 0.805 | 0.990 | 0.857 | - | - | - | - | - | - | - | - | 0.944 | 0.975 |
|  | 70% | Frequency | 0 | 2 | 9 | 22 | 4 | 963 | 0 | 0 | 0 | 0 | 0 | 0 | 0 | 0 | 0 |  | 1000 |
|  |  | Sensitivity | - | 0.833 | 0.907 | 0.864 | 0.792 | 1.000 | - | - | - | - | - | - | - | - | - | 0.995 | 0.998 |
|  |  | PPV | - | 0.833 | 0.929 | 0.783 | 0.958 | 0.987 | - | - | - | - | - | - | - | - | - | 0.982 | 0.988 |

% of cens, percentage of censoring; PPV, positive predictive value.

Cells most often selected as the optimal MRCS are shaded in grey.

Table A8. Simulation results for cluster model D (one elliptic cluster, 10% of total area) with a mean survival time of 7

|  | | | Maximum reported cluster size (MRCS) | | | | | | | | | | | | | | | | Default Setting |
| --- | --- | --- | --- | --- | --- | --- | --- | --- | --- | --- | --- | --- | --- | --- | --- | --- | --- | --- | --- |
|  | % of cens |  | 3% | 4% | 5% | 6% | 8% | 10% | 12% | 15% | 20% | 25% | 30% | 35% | 40% | 45% | 50% | Overall |  |
| Circular window | 10% | Frequency | 4 | 10 | 3 | 12 | 33 | 13 | 6 | 18 | 40 | 27 | 26 | 19 | 18 | 12 | 11 |  | 252 |
|  |  | Sensitivity | 0.000 | 0.217 | 0.389 | 0.486 | 0.520 | 0.539 | 0.583 | 0.769 | 0.775 | 0.920 | 0.930 | 0.939 | 0.972 | 1.000 | 1.000 | 0.750 | 0.201 |
|  |  | PPV | 0.375 | 0.650 | 0.778 | 0.757 | 0.729 | 0.578 | 0.500 | 0.524 | 0.419 | 0.385 | 0.312 | 0.263 | 0.235 | 0.206 | 0.184 | 0.446 | 0.422 |
|  | 30% | Frequency | 8 | 9 | 2 | 26 | 70 | 20 | 12 | 25 | 56 | 27 | 14 | 10 | 11 | 8 | 4 |  | 302 |
|  |  | Sensitivity | 0.001 | 0.259 | 0.500 | 0.590 | 0.569 | 0.608 | 0.597 | 0.747 | 0.774 | 0.932 | 0.941 | 0.967 | 0.924 | 0.979 | 1.000 | 0.695 | 0.216 |
|  |  | PPV | 0.375 | 0.778 | 1.000 | 0.978 | 0.820 | 0.603 | 0.512 | 0.490 | 0.422 | 0.402 | 0.305 | 0.259 | 0.220 | 0.197 | 0.186 | 0.567 | 0.556 |
|  | 50% | Frequency | 8 | 5 | 0 | 19 | 104 | 7 | 11 | 37 | 59 | 35 | 9 | 18 | 11 | 4 | 14 |  | 341 |
|  |  | Sensitivity | 0.001 | 0.267 | - | 0.456 | 0.622 | 0.667 | 0.530 | 0.748 | 0.757 | 0.881 | 0.870 | 0.991 | 0.970 | 0.917 | 0.929 | 0.708 | 0.256 |
|  |  | PPV | 0.500 | 0.800 | - | 0.855 | 0.825 | 0.705 | 0.459 | 0.500 | 0.416 | 0.366 | 0.283 | 0.283 | 0.226 | 0.191 | 0.171 | 0.555 | 0.534 |
|  | 70% | Frequency | 3 | 1 | 0 | 3 | 11 | 0 | 2 | 2 | 6 | 6 | 16 | 11 | 0 | 40 | 27 |  | 128 |
|  |  | Sensitivity | 0.000 | 0.000 | - | 0.000 | 0.152 | - | 0.333 | 0.500 | 0.694 | 0.722 | 0.979 | 0.970 | - | 0.896 | 0.864 | 0.760 | 0.103 |
|  |  | PPV | 0.000 | 0.000 | - | 0.000 | 0.191 | - | 0.286 | 0.338 | 0.348 | 0.271 | 0.318 | 0.257 | - | 0.189 | 0.168 | 0.211 | 0.194 |
| Elliptic window | 10% | Frequency | 1 | 9 | 9 | 6 | 21 | 22 | 27 | 36 | 37 | 29 | 24 | 24 | 17 | 12 | 9 |  | 283 |
|  |  | Sensitivity | 0.001 | 0.222 | 0.444 | 0.445 | 0.579 | 0.705 | 0.796 | 0.847 | 0.842 | 0.943 | 0.896 | 0.958 | 1.000 | 1.000 | 1.000 | 0.810 | 0.236 |
|  |  | PPV | 1.000 | 0.667 | 0.889 | 0.681 | 0.795 | 0.724 | 0.676 | 0.591 | 0.444 | 0.395 | 0.303 | 0.274 | 0.238 | 0.209 | 0.182 | 0.499 | 0.487 |
|  | 30% | Frequency | 5 | 7 | 11 | 4 | 34 | 57 | 51 | 60 | 42 | 30 | 12 | 13 | 9 | 4 | 7 |  | 346 |
|  |  | Sensitivity | 0.001 | 0.310 | 0.439 | 0.583 | 0.593 | 0.789 | 0.833 | 0.830 | 0.821 | 0.956 | 0.931 | 0.949 | 0.944 | 0.958 | 0.976 | 0.786 | 0.276 |
|  |  | PPV | 0.300 | 0.929 | 0.864 | 0.875 | 0.837 | 0.820 | 0.705 | 0.580 | 0.442 | 0.407 | 0.329 | 0.262 | 0.227 | 0.178 | 0.182 | 0.608 | 0.602 |
|  | 50% | Frequency | 2 | 3 | 7 | 3 | 28 | 58 | 83 | 121 | 45 | 25 | 23 | 8 | 16 | 8 | 12 |  | 442 |
|  |  | Sensitivity | 0.000 | 0.222 | 0.333 | 0.278 | 0.566 | 0.773 | 0.880 | 0.942 | 0.793 | 0.860 | 0.884 | 0.917 | 0.958 | 1.000 | 0.958 | 0.840 | 0.376 |
|  |  | PPV | 0.250 | 0.667 | 0.667 | 0.556 | 0.777 | 0.805 | 0.756 | 0.653 | 0.439 | 0.356 | 0.297 | 0.251 | 0.228 | 0.201 | 0.180 | 0.597 | 0.593 |
|  | 70% | Frequency | 5 | 2 | 0 | 2 | 5 | 9 | 4 | 5 | 12 | 10 | 18 | 15 | 20 | 18 | 25 |  | 150 |
|  |  | Sensitivity | 0.000 | 0.000 | - | 0.000 | 0.067 | 0.278 | 0.167 | 0.700 | 0.472 | 0.683 | 0.704 | 0.922 | 0.958 | 0.898 | 0.960 | 0.702 | 0.108 |
|  |  | PPV | 0.000 | 0.000 | - | 0.000 | 0.080 | 0.278 | 0.143 | 0.453 | 0.259 | 0.269 | 0.226 | 0.249 | 0.235 | 0.191 | 0.179 | 0.213 | 0.214 |

% of cens, percentage of censoring; PPV, positive predictive value.

Table A9. Simulation results for cluster model E (one elliptic cluster, 30% of total area) with a mean survival time of 2

|  | | | Maximum reported cluster size (MRCS) | | | | | | | | | | | | | | | | Default Setting |
| --- | --- | --- | --- | --- | --- | --- | --- | --- | --- | --- | --- | --- | --- | --- | --- | --- | --- | --- | --- |
|  | % of cens |  | 3% | 4% | 5% | 6% | 8% | 10% | 12% | 15% | 20% | 25% | 30% | 35% | 40% | 45% | 50% | Overall |  |
| Circular window | 10% | Frequency | 0 | 3 | 48 | 41 | 62 | 72 | 85 | 154 | 219 | 91 | 7 | 132 | 86 | 0 | 0 |  | 1000 |
|  |  | Sensitivity | - | 0.867 | 0.905 | 0.857 | 0.830 | 0.873 | 0.882 | 0.880 | 0.858 | 0.891 | 0.900 | 0.902 | 0.900 | - | - | 0.878 | 0.742 |
|  |  | PPV | - | 1.000 | 1.000 | 0.997 | 0.930 | 0.985 | 0.990 | 0.946 | 0.837 | 0.845 | 0.736 | 0.781 | 0.780 | - | - | 0.886 | 0.918 |
|  | 30% | Frequency | 0 | 1 | 96 | 142 | 81 | 62 | 87 | 186 | 144 | 83 | 1 | 54 | 63 | 0 | 0 |  | 1000 |
|  |  | Sensitivity | - | 0.900 | 0.868 | 0.848 | 0.769 | 0.849 | 0.874 | 0.860 | 0.828 | 0.885 | 0.900 | 0.885 | 0.898 | - | - | 0.853 | 0.726 |
|  |  | PPV | - | 1.000 | 1.000 | 0.999 | 0.894 | 0.982 | 0.988 | 0.983 | 0.872 | 0.910 | 0.720 | 0.778 | 0.779 | - | - | 0.934 | 0.955 |
|  | 50% | Frequency | 0 | 0 | 24 | 20 | 99 | 92 | 111 | 318 | 113 | 50 | 2 | 86 | 85 | 0 | 0 |  | 1000 |
|  |  | Sensitivity | - | - | 0.829 | 0.840 | 0.760 | 0.855 | 0.854 | 0.861 | 0.828 | 0.889 | 0.800 | 0.896 | 0.898 | - | - | 0.852 | 0.703 |
|  |  | PPV | - | - | 1.000 | 0.995 | 0.889 | 0.992 | 0.992 | 0.971 | 0.885 | 0.923 | 0.842 | 0.782 | 0.781 | - | - | 0.924 | 0.947 |
|  | 70% | Frequency | 0 | 0 | 6 | 30 | 51 | 39 | 35 | 568 | 43 | 5 | 2 | 221 | 0 | 0 | 0 |  | 1000 |
|  |  | Sensitivity | - | - | 0.808 | 0.790 | 0.713 | 0.795 | 0.807 | 0.849 | 0.813 | 0.820 | 0.850 | 0.906 | - | - | - | 0.847 | 0.712 |
|  |  | PPV | - | - | 1.000 | 0.996 | 0.944 | 1.000 | 0.994 | 1.000 | 0.890 | 0.830 | 0.773 | 0.780 | - | - | - | 0.942 | 0.945 |
| Elliptic window | 10% | Frequency | 0 | 0 | 0 | 1 | 3 | 36 | 25 | 429 | 169 | 5 | 266 | 66 | 0 | 0 | 0 |  | 1000 |
|  |  | Sensitivity | - | - | - | 0.850 | 0.850 | 0.860 | 0.878 | 0.905 | 0.930 | 0.800 | 0.898 | 0.946 | - | - | - | 0.907 | 0.895 |
|  |  | PPV | - | - | - | 0.895 | 0.882 | 0.895 | 0.763 | 0.910 | 0.815 | 0.957 | 0.998 | 0.904 | - | - | - | 0.913 | 0.992 |
|  | 30% | Frequency | 0 | 0 | 0 | 1 | 3 | 29 | 16 | 388 | 196 | 24 | 313 | 30 | 0 | 0 | 0 |  | 1000 |
|  |  | Sensitivity | - | - | - | 0.900 | 0.867 | 0.859 | 0.834 | 0.874 | 0.925 | 0.771 | 0.900 | 0.940 | - | - | - | 0.891 | 0.881 |
|  |  | PPV | - | - | - | 1.000 | 0.928 | 0.908 | 0.825 | 0.871 | 0.856 | 0.973 | 0.998 | 0.905 | - | - | - | 0.912 | 0.996 |
|  | 50% | Frequency | 0 | 0 | 0 | 0 | 9 | 11 | 27 | 182 | 165 | 19 | 567 | 20 | 0 | 0 | 0 |  | 1000 |
|  |  | Sensitivity | - | - | - | - | 0.878 | 0.800 | 0.832 | 0.879 | 0.927 | 0.768 | 0.900 | 0.948 | - | - | - | 0.896 | 0.894 |
|  |  | PPV | - | - | - | - | 0.976 | 0.931 | 0.734 | 0.852 | 0.868 | 0.978 | 0.999 | 0.905 | - | - | - | 0.940 | 0.997 |
|  | 70% | Frequency | 0 | 0 | 0 | 0 | 0 | 1 | 2 | 89 | 67 | 0 | 840 | 1 | 0 | 0 | 0 |  | 1000 |
|  |  | Sensitivity | - | - | - | - | - | 0.900 | 0.775 | 0.899 | 0.908 | - | 0.900 | 1.000 | - | - | - | 0.900 | 0.900 |
|  |  | PPV | - | - | - | - | - | 0.900 | 0.913 | 0.871 | 0.937 | - | 1.000 | 0.909 | - | - | - | 0.984 | 1.000 |

% of cens, percentage of censoring; PPV, positive predictive value.

Cells most often selected as the optimal MRCS are shaded in grey.

Table A10. Simulation results for cluster model E (one elliptic cluster, 30% of total area) with a mean survival time of 7

|  | | | Maximum reported cluster size (MRCS) | | | | | | | | | | | | | | | | Default Setting |
| --- | --- | --- | --- | --- | --- | --- | --- | --- | --- | --- | --- | --- | --- | --- | --- | --- | --- | --- | --- |
|  | % of cens |  | 3% | 4% | 5% | 6% | 8% | 10% | 12% | 15% | 20% | 25% | 30% | 35% | 40% | 45% | 50% | Overall |  |
| Circular window | 10% | Frequency | 12 | 4 | 0 | 12 | 26 | 29 | 31 | 64 | 61 | 56 | 57 | 159 | 163 | 43 | 63 |  | 780 |
|  |  | Sensitivity | 0.005 | 0.088 | - | 0.163 | 0.239 | 0.338 | 0.350 | 0.481 | 0.492 | 0.584 | 0.688 | 0.841 | 0.872 | 0.880 | 0.917 | 0.684 | 0.550 |
|  |  | PPV | 1.000 | 0.750 | - | 1.000 | 0.955 | 0.959 | 0.929 | 0.964 | 0.877 | 0.790 | 0.749 | 0.760 | 0.714 | 0.618 | 0.555 | 0.781 | 0.745 |
|  | 30% | Frequency | 26 | 2 | 4 | 7 | 21 | 19 | 42 | 134 | 126 | 128 | 43 | 137 | 72 | 17 | 41 |  | 819 |
|  |  | Sensitivity | 0.010 | 0.100 | 0.138 | 0.143 | 0.231 | 0.313 | 0.345 | 0.453 | 0.500 | 0.589 | 0.690 | 0.849 | 0.868 | 0.829 | 0.896 | 0.593 | 0.507 |
|  |  | PPV | 1.000 | 0.833 | 0.875 | 0.929 | 1.000 | 0.965 | 0.941 | 0.970 | 0.898 | 0.823 | 0.745 | 0.764 | 0.708 | 0.585 | 0.534 | 0.836 | 0.808 |
|  | 50% | Frequency | 23 | 0 | 1 | 9 | 18 | 6 | 28 | 96 | 117 | 129 | 22 | 252 | 88 | 25 | 56 |  | 870 |
|  |  | Sensitivity | 0.013 | - | 0.150 | 0.178 | 0.225 | 0.300 | 0.325 | 0.416 | 0.474 | 0.593 | 0.709 | 0.834 | 0.876 | 0.842 | 0.905 | 0.648 | 0.572 |
|  |  | PPV | 1.000 | - | 1.000 | 1.000 | 1.000 | 0.944 | 0.931 | 0.939 | 0.880 | 0.828 | 0.766 | 0.767 | 0.717 | 0.600 | 0.528 | 0.805 | 0.789 |
|  | 70% | Frequency | 60 | 0 | 2 | 3 | 8 | 4 | 7 | 5 | 27 | 34 | 24 | 202 | 17 | 203 | 126 |  | 722 |
|  |  | Sensitivity | 0.012 | - | 0.100 | 0.100 | 0.150 | 0.238 | 0.250 | 0.280 | 0.444 | 0.565 | 0.698 | 0.860 | 0.865 | 0.832 | 0.875 | 0.723 | 0.528 |
|  |  | PPV | 0.992 | - | 0.500 | 0.400 | 0.617 | 0.762 | 0.714 | 0.689 | 0.793 | 0.741 | 0.744 | 0.757 | 0.678 | 0.591 | 0.530 | 0.684 | 0.682 |
| Elliptic window | 10% | Frequency | 3 | 4 | 2 | 5 | 10 | 21 | 15 | 33 | 82 | 83 | 150 | 159 | 156 | 74 | 58 |  | 855 |
|  |  | Sensitivity | 0.002 | 0.088 | 0.150 | 0.200 | 0.225 | 0.286 | 0.340 | 0.444 | 0.581 | 0.714 | 0.835 | 0.879 | 0.933 | 0.942 | 0.925 | 0.784 | 0.674 |
|  |  | PPV | 1.000 | 0.667 | 1.000 | 0.950 | 0.917 | 0.925 | 0.889 | 0.951 | 0.932 | 0.927 | 0.915 | 0.825 | 0.762 | 0.667 | 0.572 | 0.829 | 0.817 |
|  | 30% | Frequency | 7 | 4 | 4 | 11 | 7 | 24 | 14 | 83 | 139 | 154 | 187 | 110 | 78 | 32 | 31 |  | 885 |
|  |  | Sensitivity | 0.006 | 0.113 | 0.138 | 0.196 | 0.214 | 0.267 | 0.321 | 0.440 | 0.575 | 0.673 | 0.847 | 0.841 | 0.907 | 0.914 | 0.923 | 0.695 | 0.620 |
|  |  | PPV | 1.000 | 0.750 | 0.875 | 0.977 | 0.914 | 0.904 | 0.889 | 0.968 | 0.957 | 0.914 | 0.928 | 0.791 | 0.729 | 0.645 | 0.559 | 0.875 | 0.862 |
|  | 50% | Frequency | 10 | 4 | 4 | 7 | 11 | 17 | 18 | 80 | 141 | 147 | 161 | 137 | 80 | 56 | 45 |  | 918 |
|  |  | Sensitivity | 0.009 | 0.100 | 0.100 | 0.214 | 0.218 | 0.274 | 0.319 | 0.426 | 0.543 | 0.654 | 0.816 | 0.838 | 0.903 | 0.935 | 0.904 | 0.690 | 0.638 |
|  |  | PPV | 1.000 | 0.667 | 0.625 | 1.000 | 0.905 | 0.922 | 0.901 | 0.953 | 0.935 | 0.907 | 0.901 | 0.778 | 0.725 | 0.664 | 0.561 | 0.847 | 0.838 |
|  | 70% | Frequency | 28 | 5 | 1 | 4 | 8 | 0 | 5 | 7 | 22 | 41 | 56 | 63 | 192 | 147 | 223 |  | 802 |
|  |  | Sensitivity | 0.008 | 0.100 | 0.100 | 0.138 | 0.144 | - | 0.230 | 0.357 | 0.509 | 0.622 | 0.709 | 0.838 | 0.858 | 0.883 | 0.976 | 0.808 | 0.652 |
|  |  | PPV | 1.000 | 0.667 | 0.667 | 0.625 | 0.588 | - | 0.596 | 0.771 | 0.862 | 0.809 | 0.761 | 0.752 | 0.687 | 0.629 | 0.604 | 0.684 | 0.682 |

% of cens, percentage of censoring; PPV, positive predictive value.

Cells most often selected as the optimal MRCS are shaded in grey.

Table A11. Simulation results for cluster model F (one irregular cluster, 20% of total area) with a mean survival time of 2

|  | | | Maximum reported cluster size (MRCS) | | | | | | | | | | | | | | | | Default Setting |
| --- | --- | --- | --- | --- | --- | --- | --- | --- | --- | --- | --- | --- | --- | --- | --- | --- | --- | --- | --- |
|  | % of cens |  | 3% | 4% | 5% | 6% | 8% | 10% | 12% | 15% | 20% | 25% | 30% | 35% | 40% | 45% | 50% | Overall |  |
| Circular window | 10% | Frequency | 6 | 25 | 19 | 328 | 274 | 184 | 27 | 69 | 32 | 9 | 7 | 0 | 16 | 2 | 2 |  | 1000 |
|  |  | Sensitivity | 0.910 | 0.871 | 0.899 | 0.907 | 0.866 | 0.941 | 0.909 | 0.921 | 0.928 | 0.880 | 0.934 | - | 0.995 | 1.000 | 1.000 | 0.904 | 0.842 |
|  |  | PPV | 1.000 | 0.997 | 0.939 | 0.999 | 0.967 | 0.864 | 0.821 | 0.810 | 0.665 | 0.703 | 0.496 | - | 0.496 | 0.473 | 0.613 | 0.919 | 0.925 |
|  | 30% | Frequency | 13 | 147 | 36 | 382 | 102 | 122 | 33 | 73 | 26 | 8 | 7 | 4 | 37 | 8 | 2 |  | 1000 |
|  |  | Sensitivity | 0.888 | 0.829 | 0.859 | 0.907 | 0.860 | 0.943 | 0.900 | 0.914 | 0.882 | 0.923 | 0.846 | 0.865 | 0.998 | 1.000 | 0.962 | 0.897 | 0.792 |
|  |  | PPV | 1.000 | 1.000 | 0.985 | 0.999 | 0.947 | 0.830 | 0.844 | 0.808 | 0.699 | 0.677 | 0.550 | 0.518 | 0.498 | 0.460 | 0.489 | 0.914 | 0.919 |
|  | 50% | Frequency | 0 | 308 | 27 | 251 | 109 | 152 | 29 | 58 | 15 | 4 | 3 | 2 | 39 | 3 | 0 |  | 1000 |
|  |  | Sensitivity | - | 0.723 | 0.781 | 0.902 | 0.806 | 0.914 | 0.897 | 0.894 | 0.892 | 0.904 | 0.846 | 0.846 | 1.000 | 0.974 | - | 0.838 | 0.735 |
|  |  | PPV | - | 1.000 | 0.994 | 1.000 | 0.953 | 0.890 | 0.854 | 0.811 | 0.691 | 0.653 | 0.550 | 0.524 | 0.501 | 0.443 | - | 0.933 | 0.934 |
|  | 70% | Frequency | 0 | 0 | 2 | 589 | 193 | 36 | 47 | 98 | 31 | 1 | 0 | 1 | 2 | 0 | 0 |  | 1000 |
|  |  | Sensitivity | - | - | 0.846 | 0.885 | 0.817 | 0.878 | 0.787 | 0.900 | 0.906 | 0.769 | - | 0.846 | 1.000 | - | - | 0.869 | 0.695 |
|  |  | PPV | - | - | 1.000 | 1.000 | 0.990 | 0.906 | 0.780 | 0.732 | 0.616 | 0.714 | - | 0.786 | 0.500 | - | - | 0.945 | 0.955 |
| Elliptic window | 10% | Frequency | 0 | 0 | 2 | 3 | 79 | 171 | 171 | 273 | 132 | 87 | 29 | 13 | 11 | 16 | 13 |  | 1000 |
|  |  | Sensitivity | - | - | 0.846 | 0.872 | 0.943 | 0.981 | 0.922 | 0.940 | 0.956 | 0.912 | 0.942 | 0.935 | 0.944 | 0.938 | 0.935 | 0.943 | 0.762 |
|  |  | PPV | - | - | 1.000 | 0.918 | 0.879 | 0.980 | 0.830 | 0.768 | 0.713 | 0.726 | 0.689 | 0.667 | 0.603 | 0.636 | 0.595 | 0.804 | 0.866 |
|  | 30% | Frequency | 1 | 1 | 0 | 11 | 66 | 164 | 132 | 316 | 138 | 77 | 49 | 10 | 13 | 17 | 5 |  | 1000 |
|  |  | Sensitivity | 0.846 | 0.846 | - | 0.916 | 0.946 | 0.986 | 0.913 | 0.937 | 0.937 | 0.902 | 0.928 | 0.939 | 0.923 | 0.919 | 0.954 | 0.939 | 0.741 |
|  |  | PPV | 1.000 | 1.000 | - | 0.903 | 0.902 | 0.983 | 0.840 | 0.774 | 0.731 | 0.755 | 0.779 | 0.637 | 0.652 | 0.680 | 0.589 | 0.815 | 0.899 |
|  | 50% | Frequency | 0 | 0 | 1 | 3 | 76 | 217 | 141 | 273 | 122 | 103 | 36 | 7 | 12 | 6 | 3 |  | 1000 |
|  |  | Sensitivity | - | - | 0.692 | 0.821 | 0.799 | 0.960 | 0.899 | 0.929 | 0.940 | 0.878 | 0.902 | 0.945 | 0.936 | 0.910 | 0.923 | 0.916 | 0.731 |
|  |  | PPV | - | - | 0.900 | 1.000 | 0.959 | 0.989 | 0.848 | 0.835 | 0.726 | 0.769 | 0.787 | 0.634 | 0.644 | 0.640 | 0.658 | 0.853 | 0.921 |
|  | 70% | Frequency | 0 | 0 | 1 | 337 | 40 | 59 | 418 | 70 | 41 | 34 | 0 | 0 | 0 | 0 | 0 |  | 1000 |
|  |  | Sensitivity | - | - | 0.692 | 0.844 | 0.800 | 0.819 | 0.900 | 0.876 | 0.707 | 0.862 | - | - | - | - | - | 0.861 | 0.666 |
|  |  | PPV | - | - | 1.000 | 1.000 | 0.910 | 0.869 | 0.846 | 0.784 | 0.834 | 0.774 | - | - | - | - | - | 0.894 | 0.918 |

% of cens, percentage of censoring; PPV, positive predictive value.

Cells most often selected as the optimal MRCS are shaded in grey.

Table A12. Simulation results for cluster model F (one irregular cluster, 20% of total area) with a mean survival time of 5

|  | | | Maximum reported cluster size (MRCS) | | | | | | | | | | | | | | | | Default Setting |
| --- | --- | --- | --- | --- | --- | --- | --- | --- | --- | --- | --- | --- | --- | --- | --- | --- | --- | --- | --- |
|  | % of cens |  | 3% | 4% | 5% | 6% | 8% | 10% | 12% | 15% | 20% | 25% | 30% | 35% | 40% | 45% | 50% | Overall |  |
| Circular window | 10% | Frequency | 2 | 1 | 15 | 68 | 205 | 113 | 85 | 116 | 102 | 65 | 12 | 14 | 94 | 66 | 40 |  | 998 |
|  |  | Sensitivity | 0.096 | 0.385 | 0.410 | 0.552 | 0.597 | 0.680 | 0.706 | 0.796 | 0.800 | 0.754 | 0.827 | 0.846 | 0.985 | 0.987 | 0.998 | 0.748 | 0.715 |
|  |  | PPV | 1.000 | 1.000 | 1.000 | 0.992 | 0.973 | 0.859 | 0.793 | 0.778 | 0.668 | 0.624 | 0.531 | 0.495 | 0.492 | 0.447 | 0.402 | 0.755 | 0.750 |
|  | 30% | Frequency | 1 | 2 | 23 | 82 | 151 | 78 | 72 | 114 | 99 | 69 | 20 | 22 | 112 | 61 | 77 |  | 983 |
|  |  | Sensitivity | 0.004 | 0.269 | 0.271 | 0.463 | 0.531 | 0.650 | 0.643 | 0.810 | 0.783 | 0.726 | 0.831 | 0.850 | 0.978 | 0.974 | 0.992 | 0.735 | 0.694 |
|  |  | PPV | 1.000 | 1.000 | 0.981 | 0.993 | 0.969 | 0.844 | 0.794 | 0.764 | 0.634 | 0.604 | 0.528 | 0.505 | 0.490 | 0.443 | 0.407 | 0.715 | 0.712 |
|  | 50% | Frequency | 2 | 4 | 82 | 75 | 179 | 56 | 117 | 121 | 53 | 43 | 11 | 23 | 166 | 25 | 27 |  | 984 |
|  |  | Sensitivity | 0.013 | 0.269 | 0.250 | 0.431 | 0.543 | 0.669 | 0.572 | 0.844 | 0.695 | 0.726 | 0.776 | 0.826 | 0.993 | 0.926 | 0.986 | 0.679 | 0.642 |
|  |  | PPV | 1.000 | 1.000 | 0.996 | 0.992 | 0.965 | 0.860 | 0.766 | 0.752 | 0.645 | 0.573 | 0.503 | 0.492 | 0.496 | 0.415 | 0.401 | 0.755 | 0.748 |
|  | 70% | Frequency | 12 | 0 | 25 | 19 | 326 | 97 | 239 | 23 | 55 | 32 | 1 | 5 | 47 | 13 | 103 |  | 997 |
|  |  | Sensitivity | 0.082 | - | 0.372 | 0.466 | 0.388 | 0.489 | 0.510 | 0.676 | 0.653 | 0.635 | 0.615 | 0.785 | 0.995 | 0.882 | 0.924 | 0.546 | 0.532 |
|  |  | PPV | 1.000 | - | 0.970 | 0.970 | 0.915 | 0.849 | 0.759 | 0.695 | 0.654 | 0.524 | 0.444 | 0.477 | 0.499 | 0.417 | 0.403 | 0.761 | 0.757 |
| Elliptic window | 10% | Frequency | 0 | 0 | 2 | 12 | 51 | 190 | 150 | 128 | 154 | 142 | 57 | 63 | 32 | 13 | 6 |  | 1000 |
|  |  | Sensitivity | - | - | 0.462 | 0.487 | 0.695 | 0.818 | 0.766 | 0.736 | 0.741 | 0.853 | 0.919 | 0.972 | 0.986 | 0.994 | 1.000 | 0.806 | 0.731 |
|  |  | PPV | - | - | 1.000 | 1.000 | 0.982 | 0.962 | 0.928 | 0.843 | 0.818 | 0.737 | 0.625 | 0.563 | 0.505 | 0.453 | 0.403 | 0.820 | 0.816 |
|  | 30% | Frequency | 0 | 4 | 1 | 15 | 54 | 151 | 168 | 96 | 103 | 152 | 79 | 71 | 42 | 39 | 23 |  | 998 |
|  |  | Sensitivity | - | 0.212 | 0.615 | 0.456 | 0.611 | 0.783 | 0.683 | 0.660 | 0.719 | 0.858 | 0.903 | 0.961 | 0.984 | 0.990 | 0.990 | 0.786 | 0.736 |
|  |  | PPV | - | 1.000 | 1.000 | 1.000 | 0.964 | 0.959 | 0.940 | 0.872 | 0.813 | 0.744 | 0.614 | 0.560 | 0.500 | 0.448 | 0.401 | 0.793 | 0.791 |
|  | 50% | Frequency | 4 | 8 | 24 | 20 | 53 | 81 | 146 | 93 | 140 | 235 | 73 | 38 | 46 | 30 | 5 |  | 996 |
|  |  | Sensitivity | 0.077 | 0.231 | 0.269 | 0.439 | 0.544 | 0.791 | 0.663 | 0.677 | 0.701 | 0.849 | 0.884 | 0.931 | 0.983 | 0.969 | 0.954 | 0.750 | 0.713 |
|  |  | PPV | 1.000 | 1.000 | 0.972 | 1.000 | 0.935 | 0.958 | 0.918 | 0.856 | 0.823 | 0.733 | 0.618 | 0.568 | 0.496 | 0.447 | 0.393 | 0.792 | 0.794 |
|  | 70% | Frequency | 0 | 0 | 1 | 3 | 50 | 161 | 36 | 181 | 518 | 34 | 5 | 6 | 2 | 1 | 2 |  | 1000 |
|  |  | Sensitivity | - | - | 0.231 | 0.487 | 0.455 | 0.511 | 0.588 | 0.613 | 0.691 | 0.864 | 0.785 | 0.859 | 1.000 | 0.923 | 0.923 | 0.640 | 0.639 |
|  |  | PPV | - | - | 0.750 | 0.958 | 0.980 | 0.923 | 0.964 | 0.886 | 0.850 | 0.749 | 0.551 | 0.512 | 0.531 | 0.444 | 0.400 | 0.870 | 0.871 |

% of cens, percentage of censoring; PPV, positive predictive value.

Cells most often selected as the optimal MRCS are shaded in grey.
